# A highly selective and potent CXCR4 antagonist for hepatocellular carcinoma treatment

**DOI:** 10.1073/pnas.2015433118

**Published:** 2021-03-22

**Authors:** Jen-Shin Song, Chih-Chun Chang, Chien-Huang Wu, Trinh Kieu Dinh, Jiing-Jyh Jan, Kuan-Wei Huang, Ming-Chen Chou, Ting-Yun Shiue, Kai-Chia Yeh, Yi-Yu Ke, Teng-Kuang Yeh, Yen-Nhi Ngoc Ta, Chia-Jui Lee, Jing-Kai Huang, Yun-Chieh Sung, Kak-Shan Shia, Yunching Chen

**Affiliations:** ^a^Institute of Biotechnology and Pharmaceutical Research, National Health Research Institutes, Miaoli County 35053, Taiwan, Republic of China;; ^b^Institute of Biomedical Engineering and Frontier Research Center on Fundamental and Applied Sciences of Matters, National Tsing Hua University, 30013 Hsinchu, Taiwan, Republic of China

**Keywords:** hepatocellular carcinoma, CXCR4 receptor, programmed cell death 1, sorafenib, tumor microenvironment

## Abstract

The CXC chemokine receptor type 4 (CXCR4) receptor and its ligand, CXCL12, are overexpressed in various cancers and mediate tumor progression and hypoxia-mediated resistance to cancer therapy. While CXCR4 antagonists have potential anticancer effects when combined with conventional anticancer drugs, their poor potency against CXCL12/CXCR4 downstream signaling pathways and systemic toxicity had precluded clinical application. Herein, BPRCX807, known as a safe, selective, and potent CXCR4 antagonist, has been designed and experimentally realized. In in vitro and in vivo hepatocellular carcinoma mouse models it can significantly suppress primary tumor growth, prevent distant metastasis/cell migration, reduce angiogenesis, and normalize the immunosuppressive tumor microenvironment by reducing tumor-associated macrophages (TAMs) infiltration, reprogramming TAMs toward an immunostimulatory phenotype and promoting cytotoxic T cell infiltration into tumor. Although BPRCX807 treatment alone prolongs overall survival as effectively as both marketed sorafenib and anti–PD-1, it could synergize with either of them in combination therapy to further extend life expectancy and suppress distant metastasis more significantly.

## Significance

A highly selective, safe, and potent CXCR4 antagonist, BPRCX807, has been designed and experimentally validated in various hepatocellular carcinoma models. Through combination therapy, it can synergize with either a kinase (e.g., sorafenib) or checkpoint inhibitor (e.g. anti–PD-1) to augment effectiveness of current anticancer treatments. With its unique mode of action, a new anticancer strategy for preventing cell migration and metastasis is provided.

Hepatocellular carcinoma (HCC) is the most common liver cancer, accounting for ∼745,500 deaths worldwide each year (1). Sorafenib is the first-line treatment for advanced HCC; this multikinase inhibitor targets the RAF/MEK/ERK pathway and VEGFR/PDGFR, eliciting antiangiogenic effects. Despite these initial anticancer activities, sorafenib only offers a limited extension to survival time for patients with HCC as cancer metastasis and primary tumor relapse occur due to rapid sorafenib resistance (2–5). Programmed cell death 1 (PD-1) immune checkpoint inhibitors—specifically, nivolumab and pembrolizumab—have been recently approved as a second-line therapeutics after sorafenib treatment failure but the response rate remains low (6, 7). Our previous studies have demonstrated that sorafenib treatment reduces mean vessel density (MVD) and therefore elevates tumor hypoxia in HCC (8, 9). This process significantly increases chemokine (C-X-C motif) ligand 12 (CXCL12) and chemokine receptor type 4 (CXCR4) expression and activates the CXCL12/CXCR4 pathway in HCC (8, 10). CXCL12 itself activates numerous signaling pathways, including the PI3K/Akt and Ras/Raf/MAPK pathways that promote tumor progression (11–13). In addition, cancer cells overexpressing CXCR4 are prone to metastasize to distant sites where cells secrete high levels of CXCL12 (14, 15). CXCL12 is a key factor that can recruit immunosuppressive bone marrow-derived cells and thus contribute to the immunosuppressive tumor microenvironment (TME) (16). Given the oncogenic potential of CXCL12/CXCR4 signaling, blockade of the CXCL12/CXCR4 axis might therefore synergize with current standard treatments—sorafenib and immune checkpoint inhibitors such as anti–PD-1—in the context of advanced HCC (9, 17), the concept of which has been experimentally validated by the discovery of a CXCR4 antagonist, **BPRCX807**.

**Fig. 1.**
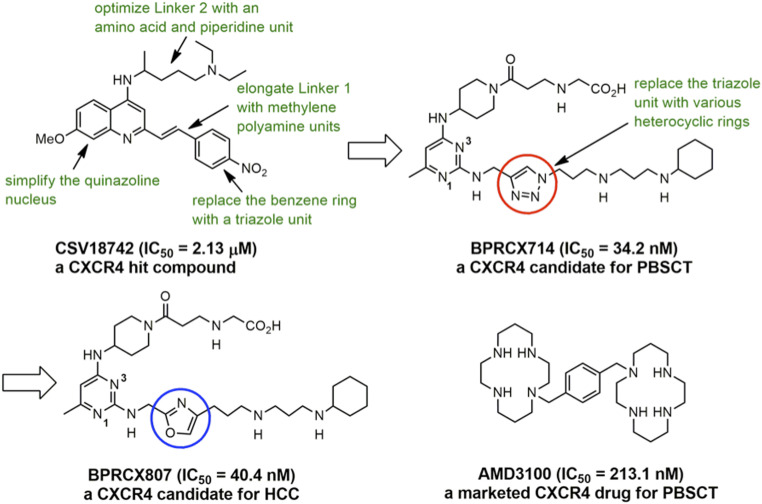
Structural evolution of the **BPRCX807** series and a current CXCR4-targeting drug.

**AMD3100** was the first Food and Drug Administration (FDA)-approved CXCR4 antagonist used for peripheral blood stem cell transplantation (PBSCT) (18); however, its application to solid tumors is limited by its poor pharmacokinetics and toxic adverse effects after long-term administration (19, 20). Thus, a CXCR4 antagonist with higher safety and better pharmacological and pharmacokinetic profiles than **AMD3100** must have great potential to serve as a clinical agent for many unmet medical-need diseases targeting CXCR4 receptors (21, 22). To this end, we initiated a new drug discovery project by screening an in-house library containing 150,000 compounds, leading to the identification of CSV18742 as a hit with an acceptable binding affinity (concentration that inhibits response by 50% [IC_50_] = 2.13 ± 0.11 µM) toward CXCR4 receptors (23). Structural modifications of this starting hit through computational docking studies (24–26) and structure-based rational design, as highlighted in green in Fig. 1, are extensively conducted. These structure–activity relationship studies are centralized on simplifying the quinazoline nucleus with a bioisosteric pyrimidine unit, optimizing the length of Linkers 1 and 2 individually located at the C2 and C4 position, and replacing a central benzene ring at Linker 1 with a triazole ring via click chemistry, accomplishing a potential candidate **BPRCX714** (IC_50_ = 34.2 ± 6.1 nM) appropriate for PBSCT (27). Based on lead **BPRCX714**, further optimization of the triazole unit through replacing it with 12 different heterocyclic five-membered rings (28, 29) was successfully implemented, culminating in **BPRCX807** (IC_50_ = 40.4 ± 8.0 nM) applicable to HCC treatment (this work). Indeed, the structural difference between **BPRCX714** and **BPRCX807** is very minor, with the former containing a triazole five-membered ring (Fig. 1, red circle) in the C2 linker and the latter characterizing an oxazole ring (Fig. 1, blue circle). Nevertheless, this subtle difference appears critical and causes a substantial impact on downstream biological effects along the CXCL12/CXCR4 signaling, details of which are presented as follows.

## Results and Discussion

### Design and Synthesis of BPRCX807.

**Scheme 1.**
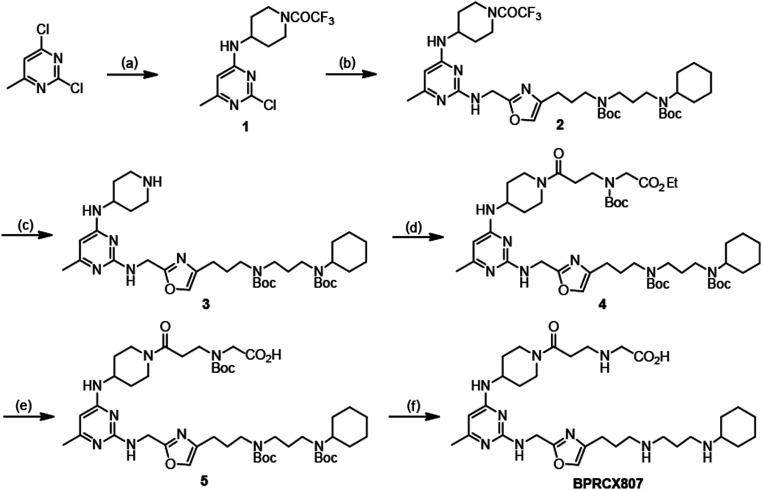
The synthesis of **BPRCX807.** (*A*) 4-amino-1-trifluoroacetyl piperidine, TEA, THF, 5 °C to rt, 16 h, 46%; (*B*) Linker 1, 2-pentanol, 140 °C, 15 h, 63%; (*C*) KOH, MeOH/THF/H_2_O, 25 °C, 16 h, 92%; (*D*) 3-(Boc (2-ethoxy-2-oxoethyl) amino)propionic acid, EDCI, HOBt, CH_2_Cl_2_, rt, 16 h, 71%; (*E*) LiOH, THF/H_2_O, rt, 16 h, 99%; (*F*) 2*N* HCl in diethylether, CH_2_Cl_2_, rt, 16 h, 95%.

The synthetic sequence of **BPRCX807** is illustrated in Scheme 1. Starting with 2,4-dichloro-6-methylpyrimidine, its more active C4-chlorine atom was first substituted with 4-amino-1-trifluoroacetyl piperidine at room temperature (rt) to produce intermediate **1** (46%), the active C2-chlorine of which was subsequently substituted with Linker 1 (30) (*SI Appendix*, Figs. S6–S10, S26, and S27) at elevated temperature (140 °C) to afford intermediate **2** in 63% yield. Intermediate **2** thus obtained was selectively hydrolyzed under basic conditions to produce **3** in high yield (92%), which in turn was coupled with 3-(Boc-(2-ethoxy-2-oxoethyl)amino) propionic acid under activation with EDCI/HOBt to furnish **4** in 71% yield. Compound **4** was subjected to basic hydrolysis to form the corresponding acid **5** in quantitative yield (99%), whose all *N*-Boc protecting groups were finally removed under acidic conditions (*2 N* HCl in Et_2_O) to achieve the desired **BPRCX807** in 95% yield as a hydrochloride salt (*SI Appendix*, Figs. S4–S6 and S28–S39).

### Preclinical Data of BPRCX807 In Vitro and In Vivo.

With **BPRCX807** in hand, extensive studies on its pharmacological, pharmacokinetic, and non-GLP (Good Laboratory Practice) toxicological profiles were then undertaken, results of which are compiled in Table 1, and each item is discussed below. Also listed is **AMD3100** as a benchmark because it is not only the solely marketed CXCR4 antagonist to date but historically has been successfully used to validate various CXCR4-mediated indications, including cancers and ischemic and inflammatory diseases, among others (10, 21, 31, 32). Biologically, the project was initially tested for binding affinity (IC_50_) and functional activity (50% effective concentration, EC_50_) toward CXCR4 receptors. **BPRCX807** (IC_50_ = 40.4 ± 8.0 nM; EC_50_ = 48.1 ± 14.4 nM; *SI Appendix*, Fig. S1) exhibited roughly fivefold stronger binding affinity than **AMD3100** (IC_50_ = 213.1 ± 26.3 nM; EC_50_ = 66.9 ± 5.5 nM) but a comparable cell-mobility activity in the chemotaxis assay, implying that their binding modes could be quite different in nature but functionally they might induce similar cell migratory efficacy. As demonstrated by releasing hematopoietic stem cells from bone marrow in mice, the number of CXCR4^+^CD34^+^ stem cells mobilized with **BPRCX807** was indeed roughly 1.5-fold higher than **AMD3100** (*SI Appendix*, Fig. S2) as reflected in their EC_50_ values (EC_50_ = 48 vs. 67 nM). Acute toxicity of **BPRCX807** was further tested following subcutaneous (SC) administration, and results indicated that its maximum tolerated dose (MTD = 75 mg/kg) was fivefold as high as **AMD3100** (MTD = 15 mg/kg, SC), suggesting that **BPRCX807** is much safer than **AMD3100** and can be used to validate many CXCR4-mediated diseases necessary for chronic treatment with emphasis on low systemic toxicity. Pharmacokinetic studies were also performed in C57BL/6 mice following SC administration, and results indicated that both maximum concentration (C_max_ = 18,833 ng/mL) and blood exposure (area under the curve [AUC] = 16,499 ng/mL·h) of **BPRCX807** are more than twofold higher than those of **AMD3100** (C_max_ = 6,200 ng/mL; AUC = 7,152 ng/mL·h; *SI Appendix*, Fig. S3), implying that it may show better in vivo efficacy in disease animal models under the same setting. Moreover, we also performed a non-GLP repeated dose toxicology of **BPRCX807** in SD rats (50 mg⋅kg^−1^⋅d^−1^, 14 d), results of which revealed that all animals survived up to study termination and all major organs, including heart, lung, kidney, liver, and so on, were normal in size, weight, and color as compared to those in vehicle (*SI Appendix*, Table S1). Meanwhile, blood sample analyses also showed that there was no significant difference in hematological and biochemical data (*SI Appendix*, Tables S2 and S3) between vehicle and tested subjects (rats). Metabolic profiles were also examined, revealing that **BPRCX807** was metabolically stable in human, mouse, rat, and dog (*SI Appendix*, Fig. S4) with no inhibitory effects on six human liver cytochrome P450 isozymes (CYP 1A2, 2C9, 2C19, 2D6, 2E1, and 3A4) up to 100 µM (*SI Appendix*, Fig. S5). As such, it is anticipated that **BPRCX807** is very likely not to cause any drug–drug interactions when coadministered with other drugs in the future. As well, a total of 67 off-target assays for **BPRCX807**, including a variety of receptors and ion channels, were also conducted (*SI Appendix*, Table S4). Results revealed that apart from exhibiting inhibition against bradykinin B1 and dopamine D3 receptor by 62% and 58%, respectively, at 10 µM, it showed very low inhibitory activities against 65 other nonprimary targets (<50% inhibition at 10 µM), suggesting that **BPRCX807** is a clean and specific CXCR4 antagonist.

**Table 1.** Preclinical profiles of BPRCX807 vs. AMD3100

Study objects	**BPRCX807**	**AMD3100**
IC_50_, nM*	40.4 ± 8.0	213.1 ± 26.3
EC_50_, nM*	48.1 ± 14.4	66.9 ± 5.5
Maximum tolerated dose, mg/kg (SC, mice, *n* = 3)^†^	75	15
C_max_, ng/mL (6 mg/kg, SC, mice, *n* = 3)*^,^^†^	18,833 ± 2499	6,200 ± 394
AUC_0–4__h_, ng/mL·h (6 mg/kg, SC, mice, *n* = 3)*^,^^†^	16,499 ± 878	7,152 ± 135
Half-life, h (6 mg/kg, SC, mice, *n* = 3)^†^	1	1
CYP450 inhibition (100 μM)^‡^	No inhibition	No inhibition
Metabolic stability in liver microsomes (mouse, rat, dog, and human)	Stable	Stable
67 off-target standard assay (inhibition >50% at 10 μM)	B1R: 62% D3R: 58%	ND^§^
*hERG* patch clamp assay	>100 μM	>100 μM
Non-GLP 14-d repeated dose (50 mg/kg, SC, rat, *n* = 5)	Clean	ND^§^

* Binding affinity or chemotaxis assay; the data are the mean values ± SD.

^†^ Pharmacokinetic studies following SC administration in C57BL/6 mice (*n* = 3).

^‡^ Cytochrome P450 tests including 1A2, 2C9, 2C19, 2D6, 2E1, and 3A4 isozymes.

^§^ Not determined.

More importantly, in the patch-clamp assay, its IC_50_ value was found to be more than 100 μM toward human K^+^ channel, suggesting that *hERG* liability, usually responsible for QT prolongation and sudden death, might not occur under treatment with **BPRCX807** (*SI Appendix*, Table S5). The specificity toward a family of chemokine receptors, including 10 CCRs, 7 CXCRs, and 1 CX3CR subtypes, through the β-arrestin assay was also conducted at 10 µM, results of which showed that **BPRCX807** displayed high selectivity for CXCR4 (100% inhibition) over other chemokine receptors (<10% inhibition), indicating that it is a functionally highly specific CXCR4-targeting antagonist (*SI Appendix*, Table S6). In summary, the above preclinical data strongly support that **BPRCX807** is a potent, safe, and target-specific drug candidate appropriate for further clinical development in many CXCR4-mediated diseases (21).

### BPRCX807 Inhibits Migration In Vitro and Suppresses Metastasis In Vivo.

**Fig. 2.**
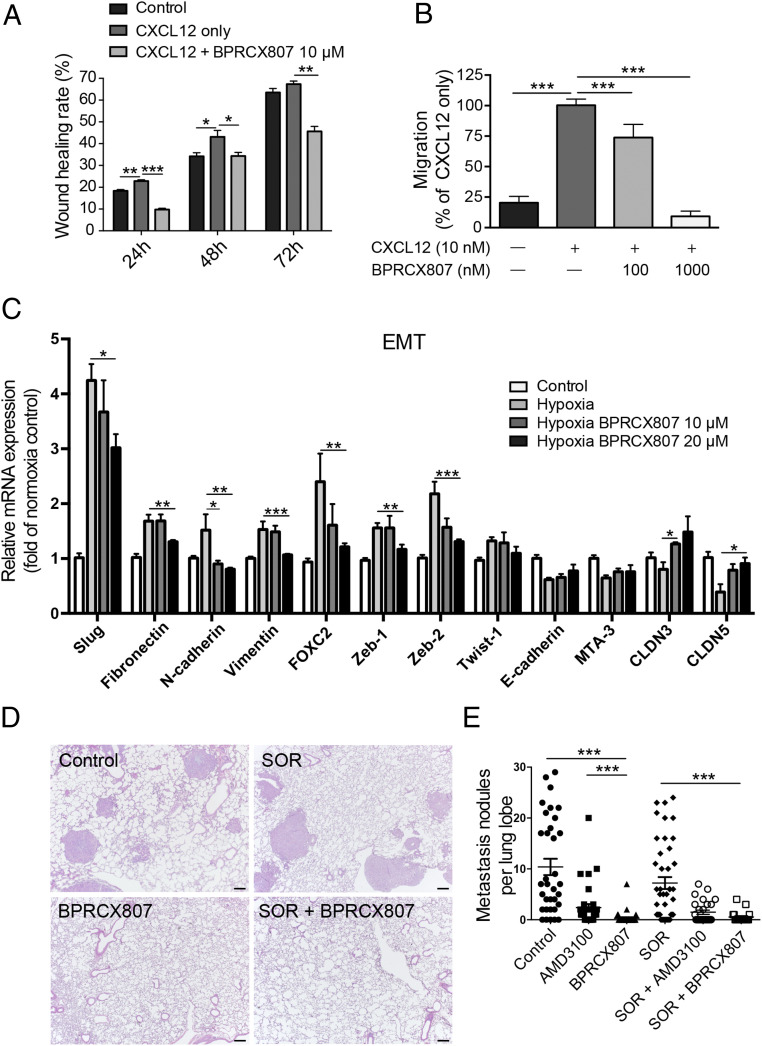
**BPRCX807** suppresses metastatic progression of HCC. (*A*) Migration assay of HCA-1 cells incubated for 24, 48, and 72 h after treatment with CXCL12 (100 ng/mL) and **BPRCX807** (10 μM) (*n* = 3). (*B*) CXCL12-induced HCA-1 chemotaxis was analyzed to measure the inhibitory activity (*n* = 3). (*C*) **BPRCX807** inhibits the EMT phenotype of HCA-1 cells under hypoxic conditions (1% oxygen). The messenger RNA levels of EMT regulators (Slug, FOXC2, Zeb-1, Zeb-2, and Twist-1), epithelial markers (E-cadherin, MTA-3, CLDN3, and CLDN5), and mesenchymal markers (vimentin, fibronectin, and N-cadherin) were determined by qRT-PCR 24 h (EMT regulators and mesenchymal markers) or 48 h (epithelial markers) after treatment with **BPRCX807** at different doses (*n* = 4 to 12). (*D*) Representative hematoxylin/eosin staining images showing metastatic tumor nodules in the lung. (Scale bars, 200 μm.) (*E*) The number of spontaneously occurring lung metastatic nodules in orthotopic HCA-1 HCC models was reduced in mice treated with **AMD3100** or **BPRCX807** (*n* = 19 to 34). The data are the mean value ± SEM. *P < 0.05, **P < 0.01, and ***P < 0.001.

Metastasis is characterized by the capability of cancer cells to invade and migrate to surrounding tissues and to establish tumors in target organs by regulating multiple signaling pathways, including the CXCL12/CXCR4 axis (12). To investigate the antimetastatic effect of **BPRCX807**, CXCL12/CXCR4 axis-mediated migration of HCC cells in vitro through wound healing and migration assays was conducted. Consequently, as HCA-1 cells were exposed to CXCL12 they exhibited faster wound closure rate than control; however, this effect was significantly suppressed upon **BPRCX807** treatment (Fig. 2*A* and *SI Appendix*, Fig. S6). Similarly, when HCA-1 cells were stimulated with CXCL12, an increase in cell migration was observed, but a significant decrease was detected upon exposure to **BPRCX807** at a concentration up to 1 µM (Fig. 2*B*). These results indicate that **BPRCX807** can effectively inhibit CXCL12/CXCR4 axis-mediated migration of HCC cells. Migration and invasion of cancer cells are also mediated by activation of the epithelial–mesenchymal transition (EMT). The CXCL12/CXCR4 axis is known to regulate the hypoxia-induced EMT in cancer cells and to facilitate metastasis. We thus examined whether blocking the CXCL12/CXCR4 axis with **BPRCX807** could reverse the hypoxia-induced EMT in vitro. We found that **BPRCX807** restrained the increases in the expression of mesenchymal markers (including Slug, Fibronectin, N-cadherin, Vimentin, FOXC2, Zeb1, and Zeb2) in HCA-1 cells cultured under hypoxic conditions in a dose-dependent manner (Fig. 2*C* and *SI Appendix*, Fig. S7). We also found that **BPRCX807** alleviated a hypoxia-induced decrease in the epithelial markers (E-cadherin, MTA-3, CLDN3, and CLDN5) in HCA-1 cells (Fig. 2*C*). Collectively, these data imply that **BPRCX807** significantly inhibits HCC cell migration by suppressing the hypoxia-induced EMT.

**Fig. 3.**
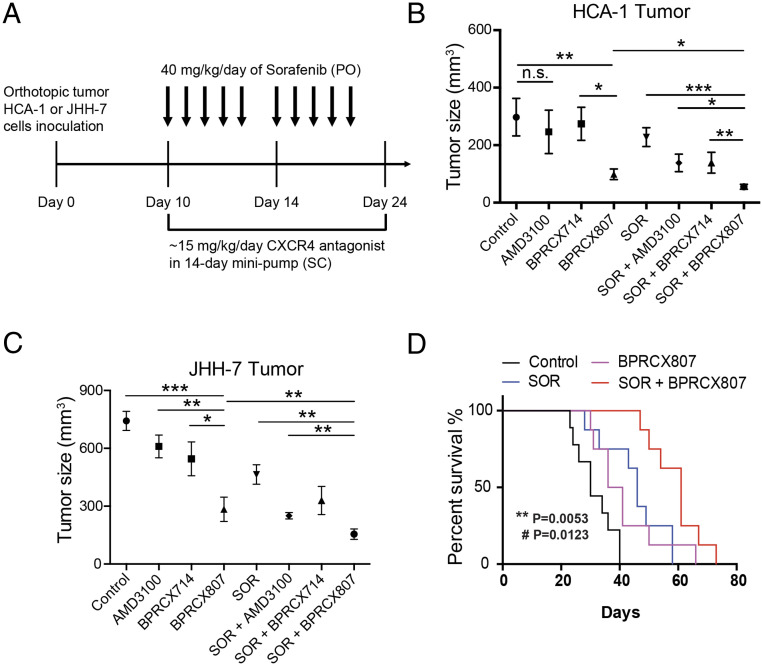
**BPRCX807** sensitizes HCC to sorafenib treatment in orthotopic HCC models. (*A*) Experimental design. Ten days after implantation of HCA-1 cells, mice were treated with **BPRCX807** using minipumps (15 mg⋅kg^−1^⋅d^−1^, SC) and with sorafenib (40 mg⋅kg^−1^⋅d^−1^, oral) five times per week, and tumor size was measured on day 24. (*B*) Tumor volumes of orthotopic HCA-1 (*n* = 10 mice for control and **BPRCX807**; *n* = 6 mice for **AMD3100**, **BPRCX714** and SOR + **BPRCX714**; *n* = 13 mice for SOR, SOR + **AMD3100** and SOR + **BPRCX807**) or (*C*) JHH-7 (*n* = 34 mice for control; *n* = 10 mice for **AMD3100**, SOR + **BPRCX807** and SOR + **BPRCX714**; *n* = 5 mice for **BPRCX714**; *n* = 7 mice for **BPRCX807**; *n* = 24 mice for SOR; *n* = 21 mice for SOR + **AMD3100**) in response to treatment with individual CXCR4 antagonists or in combination with sorafenib. The data are the mean value ± SEM. *P < 0.05, **P < 0.01, and ***P < 0.001; n.s., not significant. (*D*) The overall survival (*n* = 8) in the orthotopic HCA-1 model. **P = 0.0053, SOR plus **BPRCX807** vs. SOR alone; ^#^*P* = 0.0123, SOR plus **BPRCX807** vs. **BPRCX807** alone.

We next investigated whether suppression of the migration ability and EMT of HCC cells with **BPRCX807** can be translated to the distant metastasis in vivo. The experimental protocol is detailed in Fig. 3*A*. In brief, the orthotopic HCA-1 mouse model was established and lung metastasis would develop spontaneously within 24 d after tumor cells were implanted in liver. Histopathological analysis of tumor tissue (Fig. 2 *D* and *E*) indicated that lung metastasis is barely reduced in the sorafenib-treated group as evidenced by the number of nodules counted in lungs; however, whether given alone or combined with sorafenib, **BPRCX807** can significantly suppress lung metastasis relative to control or sorafenib-treated alone (Fig. 2*E*). Also emphasized is the fact that **BPRCX807** alone displays much better ability than **AMD3100** in lung metastasis prevention, implying that clinically it may have greater potential to become an essential element for combination cancer therapy to prevent migration and distant metastasis, a long-term issue needed to be immediately addressed in cancer treatments.

### BPRCX807 Displays Synergistic Efficacy in Combination with Antiangiogenic Therapy.

The inhibitory effects of **BPRCX807** on HCC growth in orthotopic murine HCA-1 (immunocompetent C3H mice) and human JHH-7 (immunocompromised nude mice) HCC models were further evaluated (Fig. 3*A*). **BPRCX807** and its counterparts **BPRCX714** and **AMD3100** were SC administered following osmotic minipumps by which the therapeutic cargo (15 mg⋅kg^−1^⋅d^−1^) was released constantly for 14 d since day 10; meanwhile, sorafenib (40 mg⋅kg^−1^⋅d^−1^) following the clinical setting was given orally when combination treatment was applicable. As a result, when **AMD3100** or **BPRCX714** was treated alone, they had no significant effect on tumor growth, whereas in sharp contrast **BPRCX807** treatment alone could significantly inhibit HCC growth in both human (JHH-7) and murine (HCA-1) orthotopic HCC models (Fig. 3 *B* and *C*) by reducing 67% and 62% tumor volume relative to control, respectively. More encouragingly, under combination treatment with sorafenib, the maximum suppression of HCC growth could be induced and tumor volume was shrunk by roughly 85% in the orthotopic HCA-1 model and 77% in the orthotopic JHH-7 model, indicating that **BPRCX807** could sensitize HCC cells to sorafenib and augment synergistic effects significantly. In addition, we observed that **BPRCX807** afforded at least two times more in vivo efficacy in HCA-1 than in JHH-7 mouse model; a possible explanation could be that it might serve as an immunomodulator to enhance anticancer ability of immune cells (e.g., CD8^+^ T cells) in immunocompetent mice more effectively, as seen in previous reports (9, 17). These results also motivate us to further investigate a possible mode of action on immunity associated with **BPRCX807** (discussed below). Apparently, we can conclude that **BPRCX807** is superior to its CXCR4 counterparts **AMD3100** and **BPRCX714** whether administered alone or in combination with sorafenib for antiangiogenic therapy. In addition, a long-term study on the overall survival rate was also conducted in the orthotopic HCA-1 model, results of which revealed that in combination with antiangiogenic therapy the overall survival was significantly extended compared to either **BPRCX807** (*P* = 0.0123) or sorafenib (*P* = 0.0053) alone (Fig. 3*D*).

Given that most HCCs are developed in the context of liver fibrosis and cirrhosis, to mimic clinical settings we further evaluate the effect of combination treatment in the nitrosodiethylamine (DEN)/carbon tetrachloride (CCl_4_)-induced liver fibrosis associated HCC model (*SI Appendix*, Fig. S8). Although the number of tumor nodules in mice with combination treatment was only moderately decreased compared with the control group, combination therapy can significantly reduce tumor size more efficiently than sorafenib alone, again indicating that combination treatment could confer the best therapeutic benefits on HCC models.

### BPRCX807 Reprograms the Tumor Microenvironment toward Antitumor Activity.

**Fig. 4.**
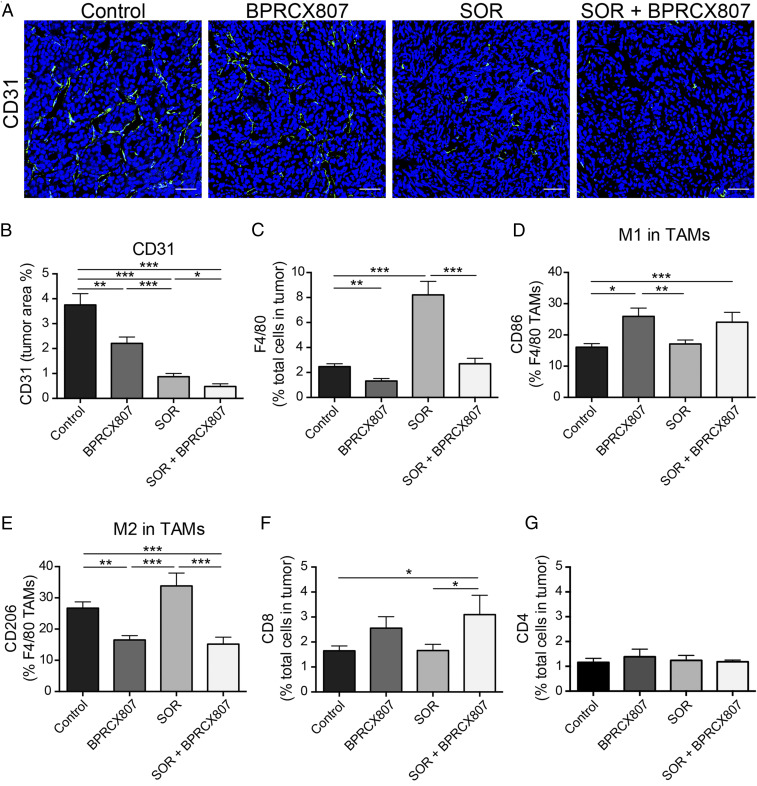
**BPRCX807** inhibits angiogenesis and remodels the immunosuppressive tumor microenvironment in the orthotopic HCA-1 model. (*A*) Representative immunofluorescence images of tumor vessels indicated by CD31^+^ staining; CD31^+^ endothelial cells were stained green and nuclei were stained blue using DAPI. (Scale bar, 50 μm.) (*B*) MVD determined by CD31^+^ staining and presented as the percentage of the total tumor area (*n* = 7 to 12). (*C*) CD45^+^F4/80^+^ TAMs. (*D*) M1-like TAMs stained by CD86. (*E*) M2-like TAMs stained by CD206. (*F*) cytotoxic CD8^+^ T cells and (*G*) helper CD4^+^ T cells were measured by flow cytometry (*n* = 9 to 17). The data are the mean value ± SEM *P < 0.05, **P < 0.01, and ***P < 0.001.

Several studies demonstrated that sorafenib could induce hypoxia via antiangiogenesis. The hypoxic TME plays a crucial role in promoting angiogenesis and metastasis as well as in suppressing antitumor immunity (9, 32–34). CXCL12/CXCR4 up-regulation in response to hypoxic stimulation contributes to these malignant features of the hypoxic TME (10, 15). To confirm the suppressive effect of **BPRCX807** in combination with sorafenib on tumor angiogenesis, we assessed MVD by CD31^+^ staining in the orthotopic HCA-1 model. Ten days after orthotopic implantation of HCA-1 cells, mice were treated with **BPRCX807**, sorafenib, or **BPRCX807** in combination with sorafenib, respectively, wherein **BPRCX807** was given using minipumps (15 mg⋅kg^−1^⋅d^−1^, SC) for 14 consecutive days and sorafenib (40 mg⋅kg^−1^⋅d^−1^, oral) five times per week for 2 wk. Quantification of MVD in HCC tumors was measured on day 24. As immunofluorescence images in tumors shown in Fig. 4*A* were translated into Fig. 4*B*, it clearly demonstrated that treatment with sorafenib or **BPRCX807** alone could significantly decrease intratumoral MVD, with the former (a typical antiangiogenic agent) being stronger than the latter, and these antiangiogenic effects could be synergistically enhanced in combination treatment. Moreover, CXCL12/CXCR4 axis up-regulation in the hypoxic TME increases recruitment of protumor bone marrow-derived cells such as tumor-associated macrophages (TAMs) and tumor-associated neutrophils, resulting in immunosuppression and resistance to anticancer treatment in the context of HCC (10). Thus, we also evaluated the effects of **BPRCX807** on F4/80^+^ TAMs recruitment in orthotopic HCA-1 tumors by flow cytometry. As a result, sorafenib-treated alone substantially increased F4/80^+^ TAMs infiltration into tumor tissues but **BPRCX807**-treated alone, on the contrary, significantly decreased TAMs infiltration; moreover, sorafenib-induced TAMs infiltration was found to be remarkably suppressed by addition of **BPRCX807** in combination treatment (Fig. 4*C*). Upon further close examination on the above F4/80^+^ TAMs we found that **BPRCX807**, whether it was treated alone or combined with sorafenib, could improve the immunostimulatory M1/immunosuppressive M2 ratio by increasing the proportion of M1-like CD86^+^ TAMs (Fig. 4*D*) and decreasing that of M2-like CD206^+^ TAMs in tumors (Fig. 4*E*), thus potentiating the antitumor immune response. Also noticed is the fact that combination treatment is allowed to increase cytotoxic CD8^+^ T cell infiltration into tumors more significantly than sorafenib alone (Fig. 4*F*). None of the treatments affected the number of tumor-infiltrating CD4^+^ T cells (Fig. 4*G*). Collectively, these analyses clearly demonstrate that **BPRCX807** can inhibit angiogenesis, promote cytotoxic T cell infiltration, reduce TAMs infiltration, and reprogram TAMs polarization in the orthotopic HCA-1 model, leading to a shift in the TME from immunosuppression toward antitumor immunity.

### BPRCX807 Displays Synergistic Effects in Combination with Immunotherapy.

**Fig. 5.**
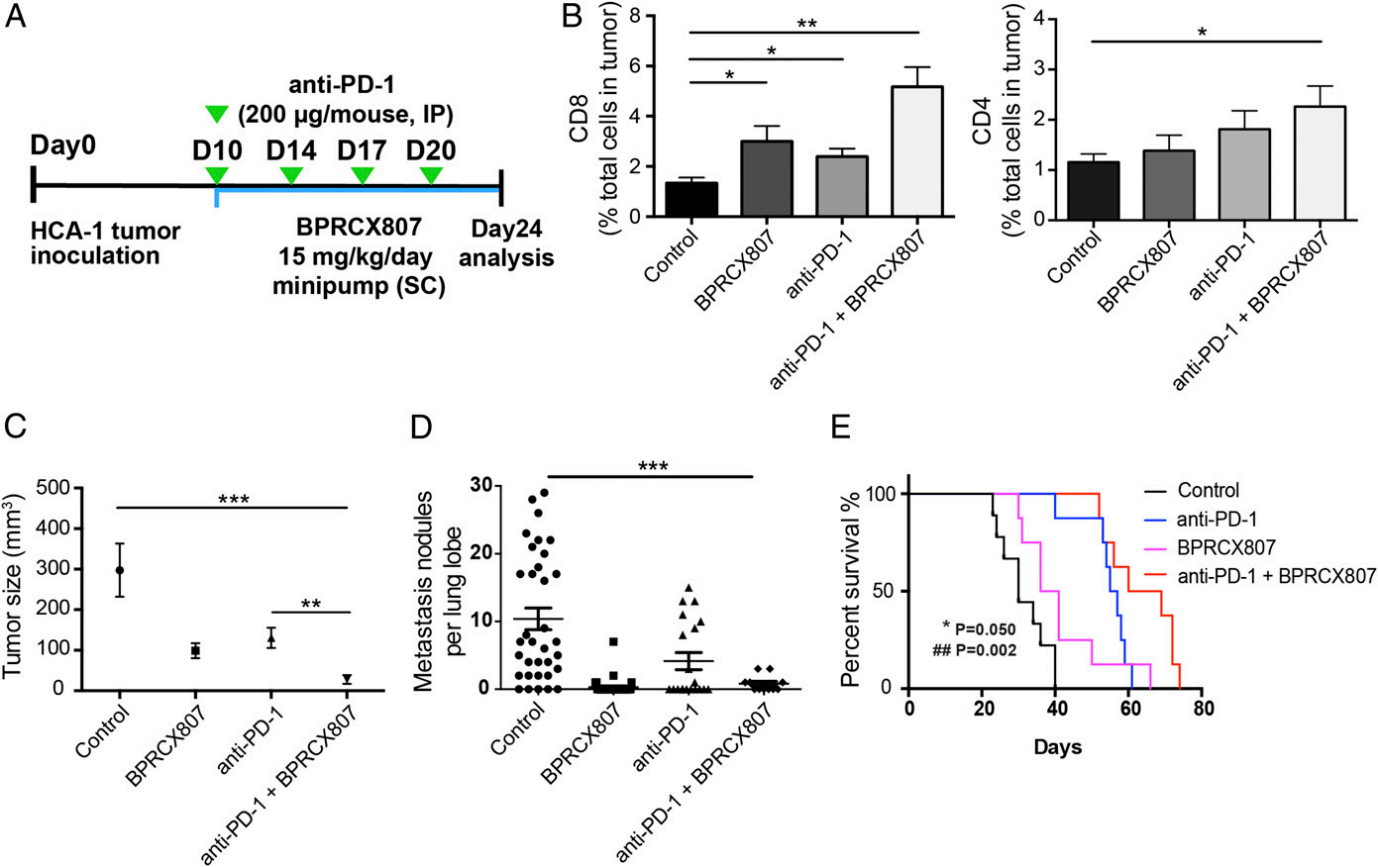
Synergistic effects of BPRCX807 with immune therapy in the orthotopic HCA-1 model. (*A*) Treatment protocol. Ten days after implantation of HCC cells, mice were treated with **BPRCX807** (15 mg⋅kg^−1^⋅d^−1^, SC) by minipump from day 10 to day 24. Anti–PD-1 Ab (200 μg/mouse) was injected IP on days 10, 14, 17, and 20 after tumor implantation. (*B*) T cells (*n* = 6 to 13) shown in CD8^+^ T cells and CD4^+^ T cells. (*C*) Tumor sizes (*n* = 10 mice for control and BPRCX807; *n* = 8 mice for anti–PD-1 and anti–PD-1 + **BPRCX807**). (*D*) Counts of lung nodules (*n* = 11 to 34). (*E*) Overall survival (*n* = 8). *P = 0.050, anti–PD-1 plus **BPRCX807** vs. anti–PD-1 alone; ^##^*P* = 0.002, anti–PD-1 plus **BPRCX807** vs. **BPRCX807** alone. The data are the mean value ± SEM. *P < 0.05, **P < 0.01, and ***P < 0.001.

Encouraged by the above positive impacts on antitumor immunity, we further evaluated whether **BPRCX807** in combination with a second-line therapeutics such as anti–PD-1, an immune checkpoint inhibitor, could increase the effectiveness of cancer immunotherapy. The experimental protocol is outlined in Fig. 5*A*. Accordingly, **BPRCX807** (15 mg⋅kg^−1^⋅d^−1^, SC) is continuously given by a minipump from days 10 to 24 after tumor implantation in the orthotopic HCA-1 model; anti–PD-1 (200 μg/mouse, intraperitoneally [IP]) is injected once on days 10, 14, 17, and 20. The study was completed and subjected to analysis on day 24. Compared to no treatment, treatment with anti–PD-1 antibody (Ab) or **BPRCX807** alone moderately increased CD8^+^ T cell infiltration into orthotopic HCA-1 tumors (Fig. 5*B*). Combined anti–PD-1 Ab and **BPRCX807** treatment, however, significantly increased CD4^+^ T and CD8^+^ T cell intratumoral infiltration (Fig. 5*B*). Consequently, in terms of efficacy as measured by tumor size reduction, treatment with **BPRCX807** alone appeared comparable to anti–PD-1, resulting in moderately inhibiting tumor growth (Fig. 5*C*); however, in sharp contrast, their combination treatment could dramatically reduce the tumor size by more than 95% vs. control (Fig. 5*C*) in the orthotopic HCA-1 model. The above remarkable outcomes might be ascribed to **BPRCX807**’s extraordinary ability to stimulate immunity by a significant increase of CD4^+^ and CD8^+^ T cells to accumulate in HCC (Fig. 5*B*). As well, lung metastasis was found to be significantly prevented in combination treatment (Fig. 5*D*) in the orthotopic HCA-1 model. Collectively, **BPRCX807** can augment antitumor immunity and sensitize HCC to anti–PD-1 treatment. More importantly, the overall survival (Fig. 5*E*) was significantly prolonged in combination treatment as compared with either anti–PD-1 or **BPRCX807** alone in the orthotopic HCA-1 model, again verifying that **BPRCX807** cannot only synergize with antiangiogenic therapy (Fig. 4) but also with immunotherapy to maximize therapeutic effects (Fig. 5).

**Fig. 6.**
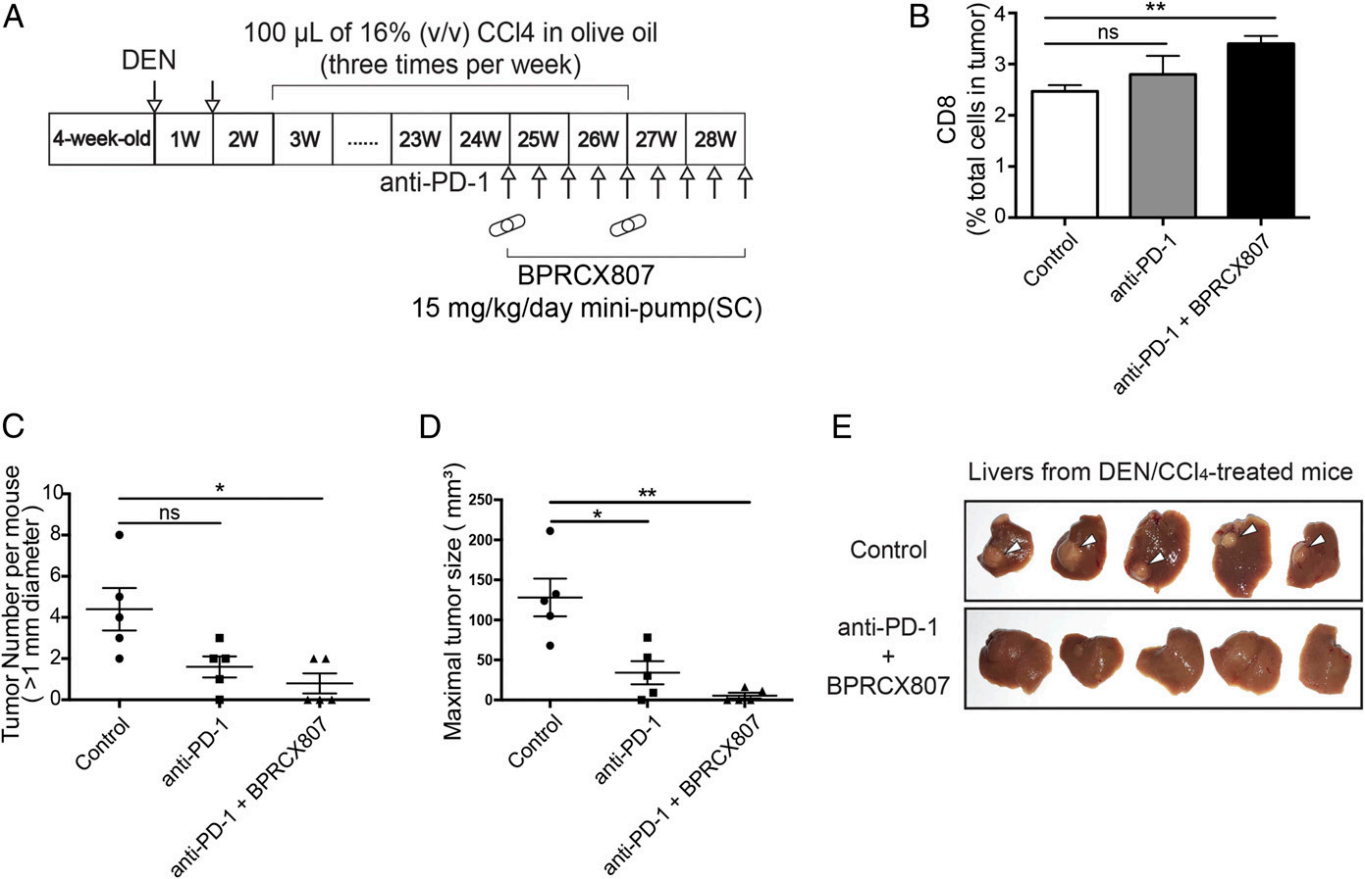
Anticancer effects of **BPRCX807** with immune therapy in DEN/CCl_4_-induced liver fibrosis associated HCC model. (*A*) Experimental protocol. (*B*) Analysis of CD8^+^ T cells (*n* = 3 mice). (*C*) Liver tumor nodule counts (>1 mm) per mouse (*n* = 5 mice). (*D*) Measurement of tumor size (*n* = 5 mice). (*E*) Representative livers in a DEN/CCl_4_-induced spontaneous HCC model; white arrows pinpoint tumor sites in fibrotic liver. The data are the mean value ± SEM. *P < 0.05 and **P < 0.01. ns, not significant.

We further evaluate the effect of combination treatment in the DEN/CCl_4_-induced liver fibrosis associated HCC model. The experimental protocol is outlined in Fig. 6*A*. **BPRCX807** was then continuously given following SC by minipumps (15 mg⋅kg^−1^⋅d^−1^) from weeks 25 to 28 along with a total of nine injections of anti–PD-1 (200 μg/mouse, IP) at an interval of 3 d. As a result, combination therapy can significantly facilitate CD8^+^ T cell tumor infiltration (Fig. 6*B*) and reduce both liver nodules (Fig. 6*C*) and tumor size (Fig. 6*D*) more efficiently than anti–PD-1 alone in the DEN/CCl_4_-induced liver fibrosis associated HCC model. More encouragingly, as pinpointed by white arrows in control, tumors in fibrotic liver were remarkably suppressed in combination treatment groups (Fig. 6*E*). In addition, **BPRCX807** is superior to its CXCR4 counterpart **AMD11070**, which is currently being studied in Phase 2/3 clinical trials in combination with immune checkpoint inhibitors, whether administered alone or in combination with anti–PD-1 (*SI Appendix*, Fig. S9) (35, 36). These outcomes again substantiate that **BPRCX807** is a perfect complement to immune checkpoint inhibitors for cancer immunotherapy. Very recently, atezolizumab (Tecentriq, PD-L1 Ab) in combination with bevacizumab (Avastin, VEGF Ab) was approved by the US FDA for the first-line treatment of patients with unresectable or metastatic HCC on the basis of superior overall survival over sorafenib (37). These encouraging clinical outcomes seem to echo that **BPRCX807**, also able to synergize with either sorafenib or anti–PD-1 effectively, might have great potential for HCC treatment in a similar fashion.

**Fig. 7.**
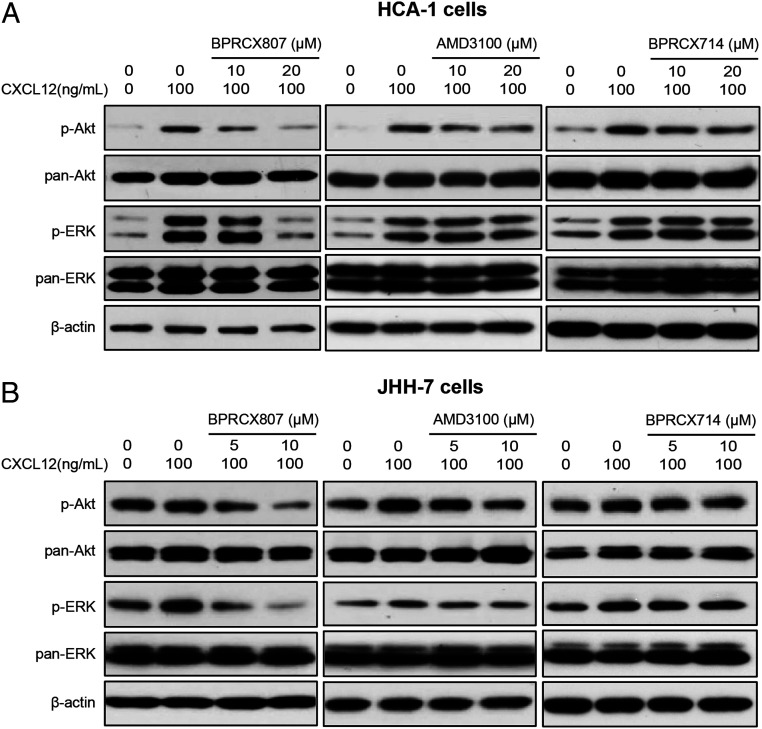
**BPRCX807** inhibited CXCL12-mediated cell signaling pathways. Western blot analysis of phospho-ERK and phospho-AKT in (*A*) HCA-1 and (*B*) JHH-7 cells.

We further assessed **BPRCX807** and its CXCR4 counterparts **BPRCX714**, **AMD3100**, and **AMD11070** in affecting downstream signaling, including ERK and Akt pathways. Accordingly, we incubated HCC cells (murine HCA-1 and human JHH-7 cells) expressing high levels of CXCR4 (8) with increasing doses of **BPRCX807** upon CXCL12 stimulation and then monitored the phosphorylation levels of Akt and ERK by Western blotting. Consequently, as indicated in Fig. 7 *A* and *B*, **BPRCX807** could suppress CXCR4/CXCL12-triggered ERK and Akt activation more significantly than its counterparts at a concentration of 20 µM in HCA-1 and 10 µM in JHH-7 cells (*SI Appendix*, Fig. S10). These results might somewhat explain why under treatment alone **BPRCX807** showed better efficacy than a typical antiangiogenic agent sorafenib in reducing tumor size in both HCA-1 and JHH-7 models (Fig. 3 *B* and *C*).

### Molecular Modeling Studies.

Considering that **BPRCX807** possesses such a distinct difference in downstream behaviors from its counterparts **BPRCX714** and **AMD3100**, computer modeling studies were then performed to look into ligand–receptor interactions at a molecular level. Instead of a traditional receptor built over the rhodopsin model by Palcewski et al. (38–40), the human CXCR4 crystal structure (RCSB Protein Data Bank ID code 4RWS) published by Wu et al. was adopted for this study (24). Most historical CXCR4 blockers are designed to contain multiple N-atoms owing to mimicking the highly positively charged nature ligand CXCL12 (23, 41–49). Based on computer modeling studies, the most stable ligand–receptor complexes I, II, and III for three test compounds are individually generated through a docking-simulation algorithm as detailed in *SI Appendix*, Figs. S12 and S13).

**Fig. 8.**
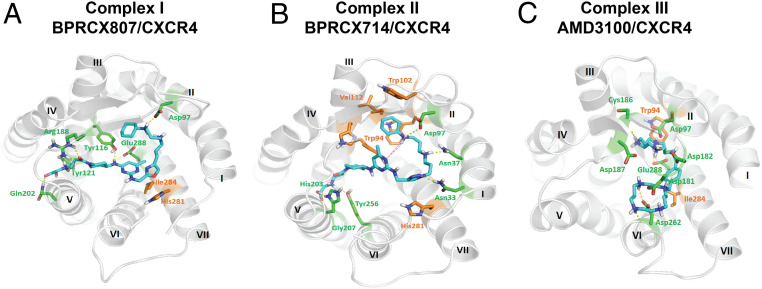
Molecular modeling studies. (*A*) Twenty-nanosecond molecular dynamics equilibrium results for **BPRCX807**, (*B*) for **BPRCX714**, and (*C*) for **AMD3100**. Hydrogen bonding is formed with residues in green as indicated by dashed lines; hydrophobic interaction is formed with residues in orange.

Complex I (Fig. 8*A*) presents the best induced-fit conformation of **BPRCX807** surrounded by Asp97, Tyr116, Tyr121, Arg188, Gln202, and Glu288 with strong hydrogen bonding, and attracted by His281 and Ile284 with strong hydrophobic interaction. This binding mode makes its terminal carboxylate insert deeply into the crevice between domains IV and V, moving the molecule toward the major subpocket built up by TMIII, IV, V, and VI. However, **BPRCX714** in complex II (Fig. 8*B*) forms hydrogen bonding with Asn33, Asn37, Asp97, His203, Gly207, and Tyr256, and van der Waals attraction with Trp94, Trp102, Val112, and His281, moving the molecule toward the minor subpocket built up by TMI, TMII, TMVI, and TMVII as proximately occupied by **AMD3100** (Fig. 8*C*). Apparently, an intramolecular hydrogen bond induced by N1 atom and HN-cyclohexyl in **BPRCX714** (Fig. 8*B*, pink dashed line) forces it to adopt a macrocyclic conformation similar to that of **AMD3100**. Coincidentally, we observed that both **BPRCX714** and **AMD3100** also showed many similar downstream effects in various HCC models, including antiangiogenesis and antimetastasis. The above results might imply that while the next generation of CXCR4 antagonists is pursued new chemical entities should be designed through their interactions with primary key residues in the major subpocket rather than the minor subpocket to approach or acquire a similar mode of action bestowed on **BPRCX807**.

## Conclusions

**Fig. 9.**
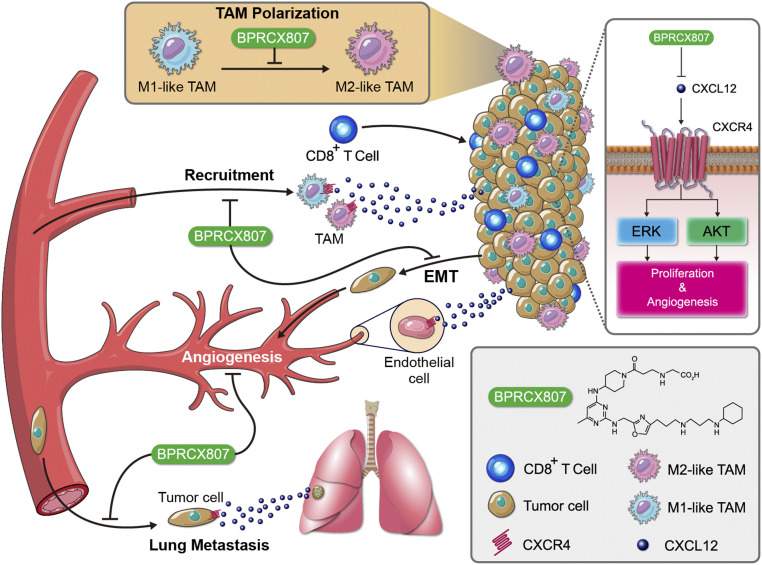
A schematic that shows the mechanism by which **BPRCX807** blocked CXCL12/CXCR4 axis–mediated cancer progression in HCC. **BPRCX807,** a highly selective, potent, metabolically stable CXCR4 antagonist, efficiently suppressed the Akt and ERK signaling pathways in HCC cells, inhibited primary tumor growth and distal metastasis, and modulated the immunosuppressive TME.

CXCR4 is highly expressed in both tumor and stromal cells in various tumor types; its overexpression is associated with poor prognosis and survival in the contexts of various cancer types. Despite the great enthusiasm for translation of CXCR4 antagonists into clinically approved cancer therapies, the utilization of these agents in solid tumors has been restricted by poor efficacy and safety concerns. These studies fully demonstrate that **BPRCX807**, a highly selective, safe, and potent CXCR4 antagonist, possesses more in vitro and in vivo efficacy than its marketed counterpart **AMD3100** under various HCC settings with supreme benefits on combination therapy, whereby it can significantly synergize with not only antiangiogenic therapy (sorafenib) but also immunotherapy (anti–PD-1) to further extend overall survival (Fig. 9). Our results suggest the clinical potential of **BPRCX807** for the treatment of HCC.

## Materials and Methods

### Cell Culture.

The murine HCC cell line HCA-1 and the human HCC cell line JHH-7 were kindly provided by Dan Duda, Massachusetts General Hospital, Boston, MA. The HCA-1 cells were maintained in high-glucose Dulbecco’s modified Eagle’s medium (DMEM; Corning). The JHH-7 cells were cultured in DME/F12 medium (Corning), and CCRF-CEM (T cell acute lymphoblastic leukemia; ATCC) cells were kept in RPMI-1640 medium (Gibco). The culture media were supplemented with 10% heat-inactivated fetal bovine serum and 1% penicillin and streptomycin (HyClone). The cells were maintained at 37 °C in an incubator (Thermo Fisher Scientific) with an atmosphere of 5% CO_2_. HEK293T cells (ATCC) were kept in DMEM (Gibco).

### Animals.

Male C3H/HeNCrNarl mice (4 to 5 wk old) and BALB/cAnN.Cg-*Foxnl*^*nu*^/CrlNarl mice (6 to 7 wk old) were purchased from the National Laboratory Animal Center (Taipei, Taiwan, Republic of China). All animals received humane care in compliance with the *Guide for the Care and Use of Laboratory Animals* (50), and all study procedures and protocols were approved by the Animal Research Committee of National Tsing-Hua University (Hsinchu, Taiwan, Republic of China) (Institutional Animal Care and Use Committee [IACUC] approval 107014). Male C57BL/6 mice, ICR mice, and SD rats were purchased from the National Laboratory Animals Center (Taipei, Taiwan, Republic of China). The animals received humane care in compliance with the *Guide for the Care and Use of Laboratory Animals* (50), and the procedures and protocols were approved by the IACUC of the National Health Research Institutes (NHRI) (Miaoli, Taiwan, Republic of China) (IACUC approval 105094).

Other experimental procedures, including synthesis of novel compounds, biological assays, animal models, and computer modeling, are described in detail in *SI Appendix*. Compound characterization date and ^1^H & ^13^C NMR spectra of key intermediates and final target **BPRCX807** are also provided.

## Supplementary Material

Supplementary File

## Data Availability

All study data are included in the article and/or supporting information.
