# Supplementary material for: A highly selective and potent CXCR4 antagonist for hepatocellular carcinoma treatment
Source: Proc Natl Acad Sci U S A. 2021 Mar 22;118(13):e2015433118. doi: 10.1073/pnas.2015433118 (PMC8020795; doi:10.1073/pnas.2015433118)
Supplement: Supplementary File [file pnas.2015433118.sapp.pdf]

Supplementary Information

A Highly Selective and Potent CXCR4 Antagonist for Hepatocellular Carcinoma Treatment

Jen-Shin Song, Chih-Chun Chang, Chien-Huang Wu, Trinh Kieu Dinh, Jiing-Jyh Jan, Kuan-Wei Huang, Ming-Chen Chou, Ting-Yun Shiue, Kai-Chia Yeh, Yi-Yu Ke, Teng-Kuang Yeh, Yen-Nhi Ngoc Ta, Chia-Jui Lee, Jing-Kai Huang, Yun-Chieh Sung, Kak-Shan Shia* and Yunching Chen*

Table of Contents

Materials and Methods ……………………………………………………………………………S3-S16

Synthetic procedures of BPRCX807 ……………………………………………………….…S4-S6

Synthetic procedures of Linker 1 ……………………………….………………..…………..S6-S10

Wound healing and invasion assays…………………………...………………….………………S10

Human CXCR4-binding assay ………………………………………………………………...S10-S11

Pharmacokinetics and toxicology studies ………………………………..…………………......S11

Cytochrome P450 testing……………..…………………………………….………………….…....S11-S12

In vivo hepatocellular carcinoma treatment studies………………….……………….…………..S12

In vivo chemical-induced HCC mice models…………..……………….………..……….............S12

Molecular docking protocol of ligand-CXCR4 receptor binding……………………..……..S12-S13

Hematoxylin and Eosin staining………………………………………………………………..S13

Flow cytometry analysis………………………………….…………………………………S13-S14

Western blot analysis…………………………………………………….……………………..S14

Reverse transcription quantitative real time PCR……………………………………………..S14-15

Off-target standard assays……………..………………………………………………………S15

hERG patch-clamp assay……………….………………………………………………….…...S15

Selectivity screening of various chemokine receptors……………………..……………….…..S15-S16

Statistics………………………………………………………………………………….……..S16

References ………………………………………..……………………………………………S17

Figure S1. Binding affinity and chemotaxis inhibition assay of BPRCX807………………………S18

Figure S2. CXCR4$^+$CD34$^+$ stem cell mobilization………………………...………………...……S18

Figure S3. The plasma concentration profile (AUC) of BPRCX807 and AMD3100 ...……………S19

Figure S4. The metabolic stabilities of BPRCX807 in liver microsomes.………….........................S19
.
Figure S5. Inhibitory values IC_{50} of BPRCX807 on 6 human liver P450 isozymes………………...S20

Figure S6. BPRCX807 suppresses CXCL12/CXCR4 axis-mediated migration of HCC cells……...S21

Figure S7. BPRCX807 inhibits the EMT phenotype of HCA-1 cells under hypoxic conditions…...S22

Figure S8. DEN/CCl4-induced liver fibrosis associated HCC model………………………………S23

Figure S9. Synergistic effects of AMD11070 with immune therapy in the orthotopic HCA-1 model……………………………………………………………………………………………...S24

Figure S10. AMD11070 moderately inhibited CXCL12-mediated cell signaling pathways………..S25

^{1}H and ^{13}C NMR spectra for all new compounds ….…………………………………………..S26-S37

Purity of BPRCX807 by HPLC analysis……………………………………….. ………… S38-S39

Table S1. Organ weight measurement of BPRCX807…...…………………………………….……S40

Table S2. Hematology analysis of BPRCX807……………………………………………………...S41

Table S3. Biochemistry analysis of BPRCX807…...…………………………………………….……S42

Table S4. 67 Off-target assays of BPRCX807…………………………….…………...………S43-S44

Table S5. *hERG* patch-clamp assay of BPRCX807………………………………….….…………..S45

Table S6. Selectivity screening of various chemokine receptors……………………………………S46

Materials and Methods

Sorafenib was purchased from Selleck Chemicals (Houston, TX). AMD3100 was purchased from Sigma Aldrich (St. Louis, MO). Anti-PD-1 was purchased from BioXcell (BE0146). AMD11070 (97% purity) in free base was purchased from ASTA Technologies (Bristol, PA), which was converted into hydrochloride salt prior to use. Unless otherwise stated, all chemical materials used were commercially available and used as supplied. Reactions requiring anhydrous conditions were performed in flame-dried glassware and cooled under an argon or nitrogen atmosphere. Unless otherwise stated, reactions were carried out under argon or nitrogen and monitored by analytical thin layer chromatography performed on glass-backed plates (5 × 10 cm) precoated with silica gel 60 F254 as supplied by Merck. Visualization of the resulting chromatograms was performed by looking under an ultraviolet lamp (λ = 254 nm) followed by dipping in an ethanol solution of vanillin (5% w/v) containing sulfuric acid (3% v/v) or phosphomolybdic acid (2.5% w/v) and charring with a heat gun. Flash chromatography was used routinely for purification and separation of product mixtures using silica gel 60 of 230−400 mesh size as supplied by Merck. Eluent systems were given in volume/volume concentrations. Melting points were determined using a KRUSS KIP1N melting point meter. ^{1}H and ^{13}C NMR spectra were recorded on a Varian Mercury-300 (300 MHz) and a Varian Mercury-400 (400 MHz). Chloroform-d, methanol-d_4 or deuterium oxide-d_2 was used as the solvent and TMS (δ 0.00 ppm) as an internal standard. Chemical shift values were reported in ppm relative to the TMS in delta (δ) units. Multiplicities are recorded as s (singlet), br s (broad singlet), d (doublet), t (triplet), q (quartet), dd (doublet of doublets), dt (doublet of triplets), and m (multiplet). Coupling constants (*J*) were expressed in Hz. Melting points were measured using an Electrothermal instrument. Electrospray mass spectra (ESMS) were recorded as *m/z* values using an Agilent 1100 MSD mass spectrometer. The purity of compounds was determined by an Agilent 1100 series HPLC system using a C18 column (Thermo Fisher 5 μm Hypersil Gold, 4.6 × 250 mm). A gradient system mixed with two eluting solvents A (water with 0.1% TFA) and B (MeOH) using a flow rate of 1 mL/min starting with 2 min at 10% solvent B, followed by a 4 min gradient of 10-50% solvent B, followed by a 10 minute gradient of 50-90% solvent B, followed by 9 min at 90% solvent B. Peaks were detected at 254 nm. IUPAC nomenclature of compounds was determined with ACD/Name Pro software.

Synthetic procedures and spectral characterization for intermediates and BPRCX807

1-[4-(2-Chloro-6-methyl-pyrimidin-4-ylamino)-piperidin-1-yl]-2,2,2-trifluoro-ethanone (1). To a magnetically stirred solution of 2,4-dichloro-6-methylpyrimidine (0.82 g, 5.0 mmol) and hydrochloride salt of 4-amino-1-trifluoroacetyl piperidine (1.21 g, 5.2 mmol) in THF (30 mL) was added TEA (1.02 g, 10.1 mmol) slowly at 5 ^{o}C. The resulting mixture was allowed to warm to room temperature and stirred for 16 h. The reaction mixture was quenched with sat. $NH_4Cl_{(aq)}$ and then extracted with ethyl acetate. The combined organic extracts were washed with brine, dried over anhydrous sodium sulfate, filtered, and concentrated. The residue thus obtained was purified by flash chromatography on silica gel with *n*-hexane/ethyl acetate (1:1) to give compound **1** (0.75 g, 46%). mp 160.2-160.7 ^{o}C. ^{1}H NMR (300 MHz, $CDCl_3$) δ 6.09 (s, 1H), 5.41 (d, *J* = 7.8 Hz, NH), 4.43 (m, 1H), 4.10 (m, 1H), 3.97 (m, 1H), 3.29 (m, 1H), 3.00 (m, 1H), 2.27 (s, 3H), 2.13 (m, 2H), 1.44 (m, 2H). ^{13}C NMR (75 MHz, $CDCl_3$) δ 167.4, 163.2, 160.5, 155.6 (q, *J* = 35.5 Hz), 116.6 (q, *J* = 286.3 Hz), 102.1, 47.4, 44.6, 42.6, 32.4, 31.5, 23.7. ESMS *m/z*: 323.1 (M+1).

[3-(*tert*-Butoxycarbonyl-cyclohexyl-amino)-propyl]-{3-[2-({4-methyl-6-[1-(2,2,2-trifluoro-acetyl)-piperidin-4-ylamino]-pyrimidin-2-ylamino}-methyl)-oxazol-4-yl]-propyl}-carbamic acid *tert*-butyl ester (2). A mixture of compound **1** (0.96 g, 3.0 mmol) and Linker 1 (1.50 g, 3.0 mmol) in 2-pentanol (45 mL) was sealed and heated at 140 ^{o}C for 15 h. The mixture was concentrated under reduced pressure to give a crude residue, which was purified by flash chromatography on silica gel with MeOH/ethyl acetate (1:9) to afford **2** (1.46 g, 63%). ^{1}H NMR (400 MHz, $CDCl_3$) δ 7.33 (s, 1H), 5.60 (s, 1H), 4.63 (d, *J* = 5.2 Hz, 2H), 4.42 (m, 1H), 3.96 (m, 1H), 3.90 (m, 1H), 3.32-2.93 (m, 8H), 2.46 (t, *J* = 7.6 Hz, 2H), 2.17 (s, 3H), 2.10 (m, 2H), 1.86-1.54 (m, 9H), 1.50-1.20 (m, 25H), 1.03 (m, 1H). ^{13}C NMR (75 MHz, $CDCl_3$) δ 165.9, 162.6, 162.2, 161.7, 155.6, 155.5, 155.5 (q, *J* = 35.5 Hz), 140.4, 134.1, 116.6 (q, *J* = 286.3 Hz), 94.6, 79.4, 79.2, 56.7 (0.34C), 55.0 (0.66C), 47.2, 46.5, 44.7, 44.1, 42.6, 40.9, 39.2, 32.6, 31.7, 31.4, 29.8, 28.6, 28.5, 26.9, 26.1, 25.6, 23.8, 23.8. ESMS *m/z*: 781.4 (M+1).

[3-(*tert*-Butoxycarbonyl-cyclohexyl-amino)-propyl]-[3-(2-{[4-methyl-6-(piperidin-4-ylamino)-pyrimidin-2-ylamino]-methyl}-oxazol-4-yl)-propyl]-carbamic acid *tert*-butyl ester (3). To a magnetically stirred solution of **2** (1.80 g, 2.3 mmol) in MeOH/THF (18 mL/18 mL) under an atmosphere of nitrogen was added a solution of KOH (0.36 g, 6.4 mmol) in H_2O (3.6 mL). The mixture was stirred at 25 ^{o}C for 16 h and then concentrated. The residue was extracted with CH_2Cl_2, and combined organic extracts were washed with brine,

dried over Na_2SO_4, filtered, and concentrated to give crude **3** (1.47 g, 92%, purity > 96%). ^{1}H NMR (300 MHz, $CDCl_3$) δ 7.32 (s, 1H), 5.60 (s, 1H), 5.41 (br s, NH), 4.63 (m, 2 H), 4.60 (br s, NH), 3.88 (m, 1H), 3.24-2.93 (m, 8H), 2.72 (m, 2H), 2.46 (t, *J* = 7.5 Hz, 2H), 2.17 (s, 3H), 2.02 (m, 2H), 1.86-1.54 (m, 9H), 1.44-1.20 (m, 25H), 1.03 (m, 1H). ^{13}C NMR (75 MHz, $CDCl_3$) δ 165.9, 162.8, 162.3, 161.7, 155.7, 155.6, 140.4, 134.1, 94.1, 79.5, 79.3, 56.8 (0.34C), 55.1 (0.66C), 48.1, 46.6, 45.4, 44.2, 40.9, 39.2, 33.3, 31.5, 29.9, 28.7, 28.6, 27.0, 26.2, 25.7, 24.0, 23.8. ESMS *m/z*: 685.4 (M+1).

(*tert*-Butoxycarbonyl-{3-[4-(2-{[4-(3-{tert-butoxycarbonyl-[3-(*tert*-butoxycarbonyl-cyclohexyl-amino)-propyl]-amino}-propyl)-oxazol-2-ylmethyl]-amino}-6-methyl-pyrimidin-4-ylamino)-piperidin-1-yl]-3-oxo-propyl}-amino)-acetic acid ethyl ester (4). To a magnetically stirred solution of crude **3** (1.41 g, 2.1 mmol,) in dichloromethane (70 mL) under an atmosphere of nitrogen were added EDCI (0.79 g, 4.1 mmol), HOBt (0.47 g, 3.1 mmol) and 3-((*tert*-butoxycarbonyl)(2-ethoxy-2-oxoethyl)amino)propionic acid (0.79 g, 2.9 mmol) sequentially in one portion. The reaction mixture was stirred at 25 ^{o}C for 16 h and then quenched with sat. $NaHCO_{3(aq)}$. The organic solution was separated and washed with brine, dried over anhydrous sodium sulfate, filtered, and concentrated. The crude residue thus obtained was purified by flash chromatography on silica gel with MeOH/ethyl acetate (7:93) to give **4** (1.38 g, 71%). ^{1}H NMR (300 MHz, $CDCl_3$) δ 7.33 (s, 1H), 5.59 (s, 1H), 5.43 (br s, NH), 4.63 (d, *J* = 5.1 Hz, 2H), 4.56 (br s, NH), 4.41 (m, 1H), 4.17 (q, *J* = 7.2 Hz, 2H), 4.01 (m, 2H), 3.89-3.86 (m, 2H), 3.58 (m, 2H), 3.26-2.96 (m, 7H), 2.79 (m, 1H), 2.67 (t, *J* = 6.6 Hz, 2H), 2.46 (t, *J* = 7.5 Hz, 2H), 2.17 (s, 3H), 2.08-1.90 (m, 4H), 1.86-1.54 (m, 7H), 1.50-1.20 (m, 37H), 1.03 (m, 1H). ^{13}C NMR (75 MHz, $CDCl_3$) δ 170.6 (0.66C, C=O), 170.4 (0.34C, C=O), 169.9 (0.66C, C=O), 169.6 (0.34C, C=O), 165.9, 162.7, 162.2, 161.7, 155.7 (0.34C, C=O), 155.6, 155.5, 155.4 (0.66C, C=O), 140.4, 134.1, 94.3, 80.5 (0.34C), 80.3 (0.66C), 79.4, 79.2, 61.0 (0.34C), 60.9 (0.66C), 56.7 (0.34C) 55.0 (0.66C), 51.2 (0.66C), 50.4 (0.34C), 47.8, 46.5, 45.6, 44.9, 44.4, 40.7, 40.7, 39.1, 32.7, 32.6, 31.9, 31.4, 29.8, 28.6, 28.5, 28.3, 26.9, 26.1, 25.6, 23.9, 23.7, 14.4. ESMS *m/z*: 942.5 (M+1).

(*tert*-Butoxycarbonyl-{3-[4-(2-{[4-(3-{*tert*-butoxycarbonyl-[3-(tert-butoxycarbonyl-cyclohexyl-amino)-propyl]-amino}-propyl)-oxazol-2-ylmethyl]-amino}-6-methyl-pyrimidin-4-ylamino)-piperidin-1-yl]-3-oxo-propyl}-amino)-acetic acid (5). To a magnetically stirred solution of **4** (1.02 g, 1.1 mmol) in THF (10 mL) under an atmosphere of nitrogen was added an aqueous solution of $LiOH_{(aq)}$ (5 mL, *1 N*). The mixture was stirred at 25 ^{o}C for 16 h and then quenched with sat. $NH_4Cl_{(aq)}$. The resulting mixture was extracted with ethyl acetate, and combined organic extracts were washed with brine, dried over

anhydrous sodium sulfate, filtered, and concentrated to give crude **5** (0.98 g, 99%, purity > 97%). ^{1}H NMR (300 MHz, CD_3OD) δ 7.63 (s, 1H), 5.75 (s, 1H), 4.60 (s, 2H), 4.40 (m, 1H), 4.01-3.90 (m, 2H), 3.81 (m, 2H), 3.56 (m, 2H), 3.28-3.02 (m, 7H), 2.81 (m, 1H), 2.68 (m, 2H), 2.48 (t, J = 7.5 Hz, 2H), 2.16 (s, 3H), 2.00-1.60 (m, 11H), 1.53-1.20 (m, 34H), 1.03 (m, 1H). ^{13}C NMR (75 MHz, $CDCl_3$) δ 175.5, 174.7 (0.34C, C=O), 174.5 (0.66C, C=O), 170.3 (0.66C, C=O), 170.0 (0.34C, C=O), 162.4, 160.7, 155.7 (0.34C, C=O), 155.6, 155.5 (0.66C, C=O), 155.4, 152.4, 140.5, 134.4, 96.5, 80.2 (0.34C), 80.1 (0.66C), 79.5, 79.3, 56.1 (0.34C), 55.0 (0.66C), 51.4 (0.66C), 51.0 (0.34C), 48.3, 46.5, 45.1, 44.8, 44.4, 40.7, 40.6, 38.4, 32.3, 31.4, 31.4, 30.7, 29.7, 28.6, 28.5, 28.3, 26.8, 26.0, 25.6, 23.6, 21.7. ESMS *m/z*: 914.5 (M+1).

(3-{4-[2-({4-[3-(3-Cyclohexylamino-propylamino)-propyl]-furan-2-ylmethyl}-amino)-6-methyl-pyrimidin-4-ylamino]-piperidin-1-yl}-3-oxo-propylamino)-acetic acid hydrochloride salt (**BPRCX807**). To a magnetically stirred solution of **5** (0.96 g, 1.05 mmol) in dichloromethane (30 mL) was added *2 N* HCl/diethyl ether (15 mL). The mixture was stirred at 25 ^{o}C for 16 h followed by filtration and concentration under reduced pressure to afford **BPRCX807** as a hydrochloride salt (4 x HCl, 0.76 g, 95%). ^{1}H NMR (400 MHz, D_2O) δ 7.69 (s, 1H), 5.90 (s, 1H), 4.73 (d, J = 17.2 Hz, 1H), 4.65 (d, J = 17.2 Hz, 1H), 4.25 (m, 1H), 3.99 (m, 1H), 3.93 (s, 2H), 3.85 (m, 1H), 3.40 (t, J = 6.4 Hz, 2H), 3.21 (m, 1H), 3.18-3.04 (m, 6H), 2.96 (t, J = 6.4 Hz, 2H), 2.84 (m, 1H), 2.61 (t, J = 7.6 Hz, 2H), 2.25 (s, 3H), 2.11-1.63 (m, 11H), 1.53-1.23 (m, 7H), 1.17 (m, 1H). ^{13}C NMR (100 MHz, D_2O) δ 169.6, 168.8, 162.9, 162.2, 153.9, 152.1, 137.1, 136.3, 97.0, 57.4, 47.7, 47.5, 46.9, 44.5, 44.2, 43.9, 41.2, 40.7, 38.3, 30.3, 29.8, 28.8, 28.6, 24.4, 24.3, 23.8, 22.8, 21.5, 17.7. ESMS *m/z*: 614.4 (M+1). purity = 98.09%, t_R = 9.17 min, The purity of BPRCX807 was determined by an Agilent 1100 series HPLC system using a C18 column (Thermo Golden, 4.6 mm × 250 mm) and the gradient system for HPLC separation was detailed in general analytical methods.

Synthetic procedures and spectral characterization of intermediates and Linker 1

The major difference between compounds BPRCX714 and BPRCX807 lies in how to construct the five-membered ring of Linker 1, of which the former is mainly achieved by the use of click chemistry[1] and the latter is synthesized by an innovative and practical sequence as outlined in Scheme S1. L-serine methyl ester hydrochloride was first coupled with dichloroacetonitrile to give chloride **L1-1** over two steps in quantitative yield (98%),[2] which was substituted with sodium azide to afford the corresponding azide **L1-2** in 83% yield. **L1-2** was further reduced with PPh_3 to yield the corresponding primary amine, which without purification was coupled with benzyl chloroformate to form Cbz-protected **L1-3** in 65% yield

over two steps. The ester functionality of intermediate **L1-3** was reduced smoothly with $NaBH_4/CaCl_2$ to afford the corresponding primary alcohol, which was subsequently oxidized with MnO_2 to produce aldehyde **L1-4** in 59% yield over two steps. Intermediate **L1-4** was subjected to Wittig reaction followed by acidic hydrolysis with 2 *N* $HCl_{(aq)}$ to furnish **L1-5** in 89% yield over two steps. Reductive amination of **L1-5** was carried out to give the corresponding amine, which was protected with Boc_2O in excess to give *N*-Boc-protected **L1-6** in 72% yield over two steps. Finally, the Cbz protecting group could be selectively removed through hydrogenolysis under standard conditions to accomplish the desired Linker **1** in 89% yield.

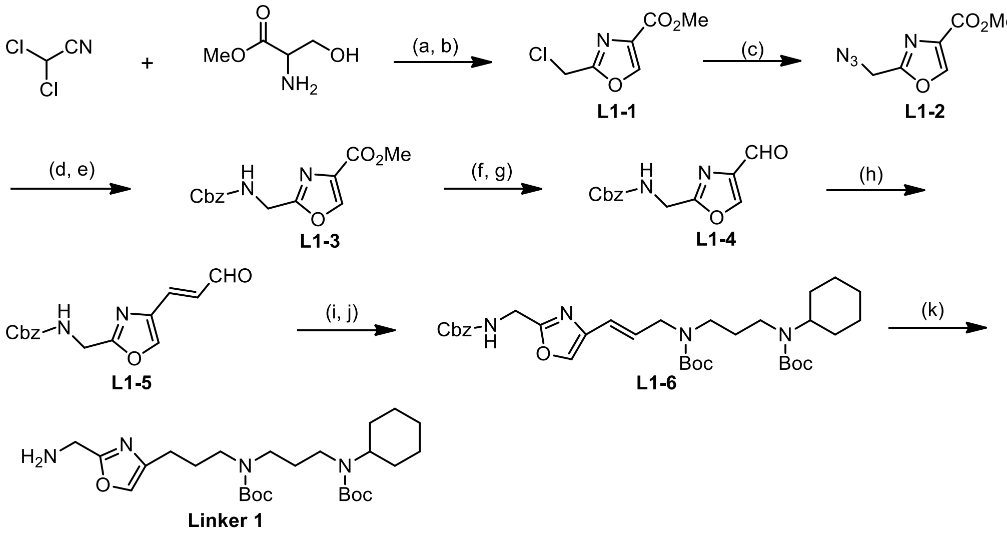

Scheme S1. The synthesis of Linker 1. (a) MeONa, CH_2Cl_2/MeOH, 0 °C, 1 h, rt, 15 h; (b) DIPEA, CH_2Cl_2, 50 °C, 15 h, 98% over two steps; (c) NaN_3, DMF, rt, 15 h, 83%; (d) PPh_3, THF/H_2O, rt, 15 h; (e) CbzCl, $NaHCO_3$, THF/H_2O, rt, 15 h, 65% over two steps; (f) $NaBH_4$, $CaCl_2$, EtOH/THF, 0 to 30 °C, 2 h; (g) MnO_2, CH_2Cl_2, rt, 2 h, 59% over two steps; (h) (1,3-dioxolan-2-ylmethyl)triphenylphosphonium bromide, *t*-BuOK, THF, 5 °C, 2 h then 2*N* HCl (aq.), 10 °C, 2 h, 89%; (i) *N*-(3-aminopropyl)cyclohexylamine, CH_2Cl_2, rt, 15 h then $NaBH_4$, MeOH, 10 °C, 1h ; (j) Boc_2O, TEA, CH_2Cl_2, rt, 3 h, 72% over two steps; (k) 10 % Pd/C, H_2, 2-propanol, 50 °C, 15 h, 89%.

2-Chloromethyl-oxazole-4-carboxylic acid methyl ester (L1-1). To a magnetically stirred solution of sodium methoxide (0.1 mL, 5.4 M in MeOH) in CH_2Cl_2 (100 mL) and MeOH (10 mL) at 0 °C was added dichloroacetonitrile (5.02 g, 45.7 mmol) over 45 min. After the solution mixture was stirred at 0 °C for 1 h, L-serine methyl ester hydrochloride (7.91 g, 50.8 mmol) was then added. The reaction mixture was stirred at 25 °C for 15 h and then quenched with water. The resulting solution was concentrated, and the residue was extracted with dichloromethane (3 x 150 mL). The combined extracts were washed with water and brine, dried over anhydrous sodium sulfate, filtered, and concentrated to give a crude residue. A

mixture of residue and DIPEA (8.91 g, 68.9 mmol) in CH_2Cl_2 (150 mL) was stirred at 50 °C for 15 h and then quenched with sat. $NH_4Cl_{(aq)}$. The aqueous phase was separated and extracted with CH_2Cl_2 (2 x 50 mL). The combined organic extracts were washed with water, brine, dried over anhydrous sodium sulfate filtered, and concentrated. The residue was purified by flash column chromatography on silica gel with *n*-hexane/ethyl acetate (4:1) to afford **L1-1** (7.85 g, 98%). mp 95.3-95.8 °C. ^{1}H NMR (300 MHz, $CDCl_3$) δ 8.25 (s, 1H), 4.64 (s, 2H), 3.93 (s, 3H). ^{13}C NMR (75 MHz, $CDCl_3$) δ 161.2, 160.0, 145.2, 133.9, 52.5, 35.4. ESMS *m/z*: 176.0 (M+1).

2-Azidomethyl-oxazole-4-carboxylic acid methyl ester (**L1-2**). A mixture of the **L1-1** (8.01 g, 45.6 mmol) and sodium azide (10.02 g, 153.8 mmol) in DMF (240 mL) was stirred at 25 °C for 15 h and then quenched with sat. $NH_4Cl_{(aq)}$. The resulting solution was extracted with Et_2O, and the combined extracts were washed with water and brine, dried over anhydrous sodium sulfate, filtered, and concentrated. The residue was purified by flash column chromatography on silica gel with *n*-hexane/ethyl acetate (4:1) to afford **L1-2** (6.91 g, 83%). mp 183.0-183.5 °C. ^{1}H NMR (400 MHz, $CDCl_3$) δ 8.25 (s, 1H), 4.46 (s, 2H), 3.90 (s, 3H). ^{13}C NMR (100 MHz, $CDCl_3$) δ 161.3, 159.7, 145.1, 133.8, 52.5, 46.6. ESMS *m/z*: 183.0 (M+1).

2-(Benzyloxycarbonylamino-methyl)-oxazole-4-carboxylic acid methyl ester (**L1-3**). A mixture of **L1-2** (6.91 g, 37.9 mmol) and triphenylphosphine (10.42 g, 39.7 mmol), in water (105 mL) /THF (210 mL) was stirred at 25 °C for 15 h. The resulting mixture was concentrated under reduced pressure to remove THF, and the residue was washed with ethyl acetate. To a mixture of amino product in the aqueous phase at 5-10 °C was added $NaHCO_3$ (6.37g, 75.8 mmol), THF (105 mL), and benzyl chloroformate (5.81 g, 34.0 mmol). The mixture was stirred at room temperature for 15 h and then quenched with aqueous NH_4Cl(aq). The resulting mixture was extracted with ethyl acetate, and the combined extracts were washed with water and brine, dried over anhydrous sodium sulfate, filtered, and concentrated. The residue was purified by flash column chromatography over silica gel with *n*-hexane/ethyl acetate (3:1) to afford **L1-3** (7.13 g, 65% over 2 steps). mp 75.7-76.2 °C. ^{1}H NMR (400 MHz, $CDCl_3$) δ 8.17 (s, 1H), 7.35-7.28 (m, 5H), 5.69 (br s, NH), 5.11 (s, 2H), 4.53 (d, J = 6.0 Hz, 2H), 3.88 (s, 3H). ^{13}C NMR (100 MHz, $CDCl_3$) δ 162.1, 161.5, 156.4, 144.5, 136.2, 133.4, 128.7, 128.4, 128.3, 67.5, 52.4, 38.4. ESMS *m/z*: 291.0 (M+1).

(4-Formyl-oxazol-2-ylmethyl)-carbamic acid benzyl ester (**L1-4**). To a solution of **L1-3** (10.02 g, 34.5 mmol) in EtOH (200 mL) and THF (100 mL) at 0-5 °C was added $CaCl_2$ (4.01 g, 36.1 mmol) and $NaBH_4$ (7.01 g, 185.3 mmol) in one portion. The resulting solution was

then stirred at 30 °C for 2 h and quenched with sat. $NH_4Cl_{(aq)}$. The solution mixture was extracted with DCM (3 x 150 mL). The combined organic extracts were washed with brine, dried over anhydrous sodium sulfate and filtered. MnO_2 (36.31 g, 417.8 mmol) was added to the filtrate and stirred at 25 °C for 2 h. The mixture was then filtered and concentrated to afford crude **L1-4**, which was subjected to chromatography purification to give **L1-4** (5.31 g, 59% over two steps). mp 78.7-79.2 °C. ^1H NMR (400 MHz, $CDCl_3$) δ 9.87 (s, 1H), 8.21 (s, 1H), 7.40-7.33 (m, 5H), 5.75 (br s, NH), 5.13 (s, 2H), 4.55 (d, *J* = 6.0 Hz, 2H). ^{13}C NMR (75 MHz, $CDCl_3$) δ 183.8, 162.8, 156.5, 145.3, 140.9, 136.1, 128.7, 128.5, 128.3, 67.5, 38.4. ESMS *m/z*: 261.0 (M+1).

[4-(3-Oxo-propenyl)-oxazol-2-ylmethyl]-carbamic acid benzyl ester (**L1-5**). A solution of **L1-4** (3.63 g, 13.9 mmol), (1,3-Dioxolan-2-ylmethyl) triphenylphosphonium bromide (7.21 g, 16.8 mmol), and *t*-BuOK (1.92 g, 17.1 mmol) in THF (100 mL) was stirred at 5 °C for 2 h. To the resulting mixture was added $HCl_{(aq)}$ (50 mL, *2 N*) and stirred at 10-15 °C for 2 h and then quenched with sat. $NaHCO_{3(aq)}$. The mixture was extracted with ethyl acetate, and the combined organic extracts were washed with brine, dried over anhydrous sodium sulfate, filtered, and concentrated. The residue was purified by flash column chromatography on silica gel with *n*-hexane/ethyl acetate (3:1) to afford product **L1-5** (3.54 g, 89%). mp 110.2-110.7 °C. ^1H NMR (400 MHz, $CDCl_3$) δ 9.66 (d, *J* = 8.0 Hz, 1H), 7.85 (s, 1H), 7.40-7.33 (m, 5H), 7.27 (d, *J* = 15.6 Hz, 1H), 6.82 (dd, *J* = 15.6, 8.0 Hz, 1H), 5.49 (br s, NH), 5.15 (s, 1H), 4.54 (d, *J* = 5.6 Hz, 1H). ^{13}C NMR (100 MHz, $CDCl_3$) δ 193.2, 162.4, 156.4, 140.4, 139.2, 137.5, 136.2, 130.5, 128.8, 128.5, 128.4, 67.6, 38.6. ESMS *m/z*: 287.1 (M+1).

{3-[2-(Benzyloxycarbonylamino-methyl)-oxazol-4-yl]-allyl}-[3-(tert-butoxycarbonyl-cyclohexyl-amino)-propyl]-carbamic acid tert-butyl ester (**L1-6**). To a magnetically stirred solution of **L1-5** (6.55 g, 22.9 mmol) in dry CH_2Cl_2 (110 mL) were added N-cyclohexyl-1,3-propanediamine (4.11 g, 26.3 mmol) and $MgSO_4$ (10.51 g) in one portion. The mixture was stirred at 25 °C for 15 h and then filtered. The filtrate was added into the solution of $NaBH_4$ (1.38 g, 36.5 mmol) in MeOH (240 mL) slowly. The mixture was stirred at 10 °C for 1 h and then concentrated. The residue was dissolved in CH_2Cl_2 (150 mL), and the organic solution was washed with NH_4Cl, brine, dried over anhydrous sodium sulfate, and filtered. Boc_2O (11.51 g, 52.7 mmol) and TEA (5.35 g, 53.0 mmol) were added sequentially in one potion to the filtrate. The mixture was stirred at room temperature for 3 h and then concentrated. The residue was purified by flash column chromatography on silica gel with *n*-hexane/ethyl acetate (1:1) to afford **L1-6** (10.35 g, 72% over 2 steps). ^1H NMR (300 MHz, $CDCl_3$) δ 7.47 (s, 1H), 7.36-7.31 (m, 5H), 6.36-6.22 (m, 2H), 5.42 (br s, NH),

5.14 (s, 2H), 4.49 (d, J = 5.7 Hz, 2H), 3.94 (m, 2H), 3.22-2.98 (m, 4H), 1.80-1.56 (m, 7H), 1.45 (s, 9H), 1.43 (s, 9H), 1.41-1.20 (m, 5H), 1.03 (m, 1H). ^{13}C NMR (75 MHz, $CDCl_3$) δ 161.0, 156.4, 155.6, 155.6, 138.6, 136.3, 135.3, 128.7, 128.4, 128.3, 119.9, 119.2, 79.8, 79.3, 67.4, 56.7 (0.34C), 55.1 (0.66C), 48.5, 44.7, 40.9, 38.6, 31.4, 29.8, 28.7, 28.6, 26.2, 25.7. ESMS *m/z*: 627.4 (M+1).

[3-(2-Aminomethyl-oxazol-4-yl)-propyl]-[3-(tert-butoxycarbonyl-cyclohexyl-amino)-propyl]-carbamic acid tert-butyl ester (**Linker 1**). A mixture of **L1-6** (10.35 g, 16.5 mmol) and 10% Pd/C (4.66 g) in *iso*-propanol (310 mL) was stirred under $H_{2(g)}$ at 50 °C for 15 h. The resulting mixture was filtered, and the filtrate was concentrated to afford Linker 1 (7.23 g, 89 %). ^{1}H NMR (300 MHz, $CDCl_3$) δ 7.31 (s, 1H), 3.90 (s, 2H), 3.23-2.92 (m, 6H), 2.43 (t, J = 7.2 Hz, 2H), 1.81-1.50 (m, 9H), 1.41 (s, 18H), 1.40-1.18 (m, 5H), 1.02 (m, 1H). ^{13}C NMR (75 MHz, $CDCl_3$) δ 164.1, 155.6, 155.6, 140.3, 134.3, 79.4, 79.3, 56.6 (0.34C), 55.1 (0.66C), 46.5, 44.8, 40.9, 39.3, 31.4, 29.7, 28.7, 28.6, 26.9, 26.1, 25.7, 23.7. ESMS *m/z*: 495.3 (M+1).

Wound healing and invasion assays

Cell migration ability was measured by wound healing assay. HCA-1 cells (5 × 10^5 cells per well) were seeded onto 12-well plates and incubated overnight. When the cell confluence exceeded 90%, scratch wounds were made by scraping a straight line in the middle of the cell layer using the tip of a 1000 μL pipette. After washing the cells with PBS, the cells were incubated in serum-free medium under different treatment conditions (control, 100 ng/mL CXCL12, and 10 μM BPRCX807 with 100 ng/mL CXCL12). Then, 3-4 fields (40×) of each group were randomly chosen and observed by a microscope (IX83, Olympus, Japan) at time points of 0 h, 24h, 48 h and 72 h. For the invasion assay, HCA-1 cells were suspended in RPMI 1640 containing 0.5% FBS and then preincubated with the indicated concentrations of compounds for 10 min at 37 °C. The assay was performed in Millicell Hanging Cell Culture Inserts (pore size 8 μm; 24-well plate; Millipore, Bedford, MA). A mixture of BPRCX807 and 10 nM CXCL12 was added to the lower chambers of the inserts, and cells with compounds were plated in the upper chambers of the inserts at a density of 2.5 × 10^5 cells/well. After 17.5 h of incubation at 37 °C, the cells in both chambers of the inserts were measured with a CellTiter-Glo Luminescent Cell Viability Assay (Promega, Wisconsin).

Human CXCR4-binding assay

Aliquots of 2.5 μg of purified HEK293T cell membrane fractions with CXCR4 were incubated with 0.16 nM [^{125}I]CXCL12 (PerkinElmer, Boston, MA) and the indicated

concentrations of BPRCX807 in incubation buffer (50 mM HEPES-NaOH, pH 7.4, 100 mM NaCl, 5 mM $MgCl_2$, 1 mM $CaCl_2$, 0.5% BSA). Nonspecific binding was defined in the presence of 50 μM AMD3100 (plerixafor). The reaction mixtures were incubated for 1.5 h at 30 °C and then transferred to a 96-well GF/B filter plate (Merck Millipore, Billerica, MA). The reaction mixtures were terminated by manifold filtration and washed with ice-cold wash buffer (50 mM HEPES-NaOH, pH 7.4, 100 mM NaCl) four times. The radioactivity bound to the filter was measured with a TopCount instrument (PerkinElmer, Waltham, MA). The IC_{50} values were determined as the concentrations of the compounds required to inhibit 50% of the specific binding of [^{125}I]CXCL12 and were calculated by nonlinear regression (GraphPad software, San Diego, CA).

Pharmacokinetics and toxicological studies

Male C57BL/6 mice, each weighing 23.4–25.4 g, were quarantined for 1 week before drug treatment. Compounds dissolved in 100% saline were individually given to mice (n = 3) subcutaneously (6 mg/kg; nonfasted mice). Blood samples were collected via cardiac puncture at defined time points (0 (immediately before dosing), 0.03, 0.08, 0.25, 0.5, 1, 2, and 4 h after dosing) and then stored at −80 °C. The volume of the dosing solution given was 100 μL for each mouse. The plasma samples were analyzed by liquid chromatography–tandem mass spectrometry (LC–MS/MS), and the data were calculated by a standard noncompartmental method using the Kinetica software program (InnaPhase). ICR mice were subcutaneously treated with BPRCX807 (n = 3/group) from a low to a high dose to determine the maximum tolerated dose (MTD) based on zero mortality (n = 3, 0/3). Test animals following administration of the target compound at its highest dose remain alive and temporary adverse effects such as decreased movement/activity can be recovered within 2 h after dosing. In a non-GLP repeated dose toxicology study, BPRCX807 solved in saline was subcutaneously administered to SD rats at 50 mg/kg/day for 14 consecutive days. The organ and body weights of the rats were examined using an electronic balance. Blood samples were collected from all animals 28 h after the last injection for serum chemistry and hematology.

Cytochrome P450 testing

The effects of BPRCX807 on the activity of six specific human liver cytochrome P450 isozymes (CYP 1A2, 2C9, 2C19, 2D6, 2E1, 3A4) expressed in baculovirus-infected insect cell supersomes were studied. The reactions used to measure the activity of these enzymes

were phenacetin O-deethylation (1A2), diclofenac 4-hydroxylation (2C9), mephenytoin 4-hydroxylation (2C19), dextromethorphan O-demethylation (2D6), chlorzoxazone 6-hydroxylation (2E1) and testosterone 6-hydroxylation (3A4). Furafylline, sulfaphenazole, tranylcypromine, quinidine, diethyldithiocarbamate and azamulin were used as reference inhibitors of these enzymes. Incubation mixtures containing no BPRCX807 or reference inhibitors were used as controls.

In vivo hepatocellular carcinoma treatment studies

To establish murine or human HCC models, HCA-1 or JHH-7 cells were orthotopically implanted in the livers of 7-week-old C3H/HeNCrNarl and 9-week-old BALB/cAnN.Cg-*Foxnl*^{nu}/CrlNarl mice separately using 20 μl of Matrigel solution.[3] The groups of HCA-1 and JHH-7 tumor-bearing mice included a control group and groups gavaged with sorafenib at a dose of 40 mg/kg in 1% Tween 80 (5 doses/week) and with one of the CXCR4 inhibitors (AMD3100, BPRCX714 and BPRCX807) (15 mg/kg/day, subcutaneously by osmotic minipump; Sigma). For combined anti-PD-1 immunotherapy, ten days after tumor implantation, mice were injected intraperitoneally four times (at 3- to 4-d intervals) with anti-mouse PD-1 (200 μg/mouse; BioXcell, BE0146). The therapeutic efficacy and the changes in immune cells were analyzed after two weeks of treatment. The tumor volume was calculated with the following formula: volume = width × length × height/2. The lung metastatic nodules were counted with a dissection microscope (SMZ-1B, Nikon, Japan).

In vivo chemical-induced HCC mice models

C3H mice were administered diethylnitrosamine (DEN; two IP injections of 1mg/kg at 4 and 5 weeks of age). Later, CCl_4 (100 μL of 16% (v/v) in olive oil, 3 times/week P.O. starting at 6 weeks of age) was administered for 24 weeks to develop chemical-induced, fibrosis-promoted HCC. Treatment of BPRCX807 in minipumps (15 mg/kg/day, SC) and anti-mouse PD-1 (200 μg/mouse; IP; BioXcell, BE0146) was initiated after 22 weeks of CCl_4 administration, and spontaneous HCC formation was examined after 4 weeks of treatment.

Molecular docking protocol of ligand-CXCR4 receptor binding

The protein structure of human CXCR4 (Protein Data Bank identifier (PDB ID): 4RWS) was applied for the docking strategy to illustrate the binding interaction with BPRCX807, BPRCX714 and AMD3100. Before docking calculation was carried out, the mutated residues

(L125W, D187C, and T240P) in the published X-ray PDB file were returned to their original residues. All docking calculations were performed by using the Discovery Studio 2018/LigandFit program (BIOVIA, Inc., San Diego, CA). Molecular dynamics (MD) simulations were carried out in GROMACS v5.1.2 software to refine the docked conformation.[4] After 20 ns simulations, the complex structure of BPRCX807, AMD3100 and BPRCX714 could attain stable and equilibrium states. The topology of each docked ligand was generated by the PRODRG server.[5] The force field for the whole system was the GROMOS 43a1 force field.[6] The protein-ligand complex was restrained in a cube whose edges were placed 1 nm from the complex, and the SPC/E water model was used. The system was electrically neutralized by adding 9 Cl^- ions. Energy minimization was performed using the steepest descent and conjugate gradient methods to converge the system to 10 kJ/mol/nm. After a short energy minimization step, the system was subjected to NVT (300 K) and NPT (1 bar) equilibration with a 100 ps running time, and the LINCS algorithm was used to constrain the hydrogen bond lengths.[7] The time step was kept at 2 fs for the simulation. A cut-off distance of 10 Å was used for all short-range nonbonded interactions, and 12 Å Fourier grid spacing was used in the PME method for long-range electrostatics. Finally, the restraints were removed, and a 20 ns MD calculation was performed.

Hematoxylin and Eosin staining

Lung tissue was cut into small pieces and fixed in 4% PFA (in PBS) overnight before being embedded in paraffin wax. The sections were then stained with hematoxylin and eosin (H&E) and observed with an inverted microscope (IX83, Olympus, Japan).

Flow cytometry analysis

HCA-1 tumor-bearing mice were anesthetized, perfused via intracardiac injection with PBS and then sacrificed. Tumor tissues were collected and harvested in enzyme-containing DMEM cell culture medium (1.5 mg/mL of collagenase type 1A and hyaluronidase; Sigma). The tissues were cut and ground into smaller pieces and then digested for 1 h at 37 °C to form suspensions of single tumor cells. The suspensions were filtered through a 70-µm cell strainer (Corning, Manassas, VA), and the cells were then incubated with rat anti-mouse CD16/CD32 mAbs to block Fc receptors. After washing 3 times and resuspending in cold flow buffer (1% BSA, 0.1% NaN_3 in PBS), the single-cell suspensions were incubated with monoclonal anti-mouse F4/80-PE (eBiosciences, San Diego, CA), 7-AAD, CD3e-APC, CD8a-PE-Cy7, CD45-FITC, CD4-PE (BD Biosciences, East Rutherford, NJ), CD86-APC-

Cy7, and CD206-APC (BioLegend, San Diego, CA) antibodies, as previously described.[8] Flow cytometry data were acquired on a BD FACSAria III flow cytometer and analyzed with FACSDiva software.

C57BL/6 male mice were treated with potential CXCR4 antagonist individually by subcutaneous injection, and then blood samples containing mobilized stem/progenitor cells were collected 2 hours later. After labeled with specific antibodies, including APC-conjugated anti-CXCR4 (clone 2B11; eBioscience) and FITC-conjugated anti-CD34 (clone RAM34; eBioscience), cells were washed, characterized and quantified by flow cytometer (Guava Technologies, Hayward, CA, USA). Each data point included at least 60,000 events for analysis of other cell types.

Western blot analysis

Western blotting was conducted by following standard procedures. HCA-1 or JHH-7 cells (10^5 cells per well) were seeded in 12-well plates (Costar, IL), incubated for 12 h and treated with AMD3100, BPRCX714 or BPRCX807 at different concentrations in rCXCL12-containing (100 ng/mL) serum-free medium. After 24 h, the cells were lysed in RIPA buffer on ice for 30 min, and samples were collected and loaded with 4× Laemmli Sample buffer (Bio-Rad, Hercules, CA). The samples were separated on a 10% SDS-PAGE gels and transferred to PVDF membranes. After blocking with skim milk for 1 h, the membranes were incubated at 4°C overnight with primary antibodies against p-AKT, AKT, p-ERK, ERK (Cell Signaling, Danvers, MA) or β-actin (Sigma-Aldrich, St. Louis, MO) before incubation with HRP-conjugated secondary antibodies (Antibodies Inc., Davis, CA) for 1 h. Autoradiography was conducted after ECL reagent (Thermo Scientific, Rockford, IL) was added to the membranes.

Reverse transcription quantitative real time PCR

Total RNA was extracted from HCA-1 cells using the RNeasy Mini Kit (Qiagen, CA, USA). cDNAs were synthesized using the High-Capicity cDNA Reverse Transcription Kit (Applied Biosystems, CA, USA). Primers specific for Slug, Fn1, Cdh2, Vim, Foxc2, Zeb1, Zeb2, Twist1, Cdh1, Mta3, Cldn3, Cldn5, β-actin and GAPDH were used, and relative gene expression was determined using Real-Time SYBR Green PCR Master Mix (Applied Biosystems, CA, USA) on a QPCR System. The comparative threshold cycle method was used to calculate fold change in gene expression, which was normalized to β-actin as a reference gene.

Table: List of genes examined by real-time PCR and the primer sequences used.

Genes	Forward primers	Reverse primers
Slug	5'- TGGTCAAGAAACATTTCAACGCC-3'	5'-GGTGAGGATCTCTGGTTTTGGTA -3'
Fn1	5'-TGTGACCAGCAACACGGTG-3'	5'-ACAACAGGAGAGTAGGGCGC-3'
Cdh2	5'-AGCGCAGTCTTACCGAAGG -3'	5'- TCGCTGCTTTCATACTGAACTTT -3'
Vim	5'-CTTGAACGGAAAGTGGAATCCT-3'	5'-GTCAGGCTTGGAAACGTCC-3'
Foxc2	5'- AACCCAACAGCAAACTTTCCC -3'	5'- GCGTAGCTCGATAGGGCAG -3'
Zeb1	5'- ACTGCAAGAAACGGTTTTCCC -3'	5'- GGCGAGGAACACTGAGATGT -3'
Zeb2	5'-ATTGCACATCAGACTTTGAGGAA-3'	5'-ATAATGGCCGTGTCGCTTCG-3'
Twist1	5'-CTGCCCTCGGACAAGCTGAG-3'	5'-CTAGTGGGACGCGGACATGG-3'
Cdh1	5'-CAGTCATAGGGAGCTGTCTACCAAA-3'	5'-GGGTACACGCTGGGAAACAT-3'
Mta3	5'-AAAAGCAGAAGCACCAGGAA-3'	5'-GGCCCATCTAGACCATTGTG-3'
Cldn3	5'- CCAACTGCGTACAAGACGAG -3'	5'- CCAGGACACCGGTACTAAGG-3'
Cldn5	5'- CTCTGCTGGTTCGCCAACA -3'	5'- CCCAGCTCGTACTTCTGTGACA -3'
β-actin	5'-TGAGAGGGAAATCGTGCGTG-3'	5'-TTGCTGATCCACATCTGCTGG-3'
GAPDH	5'-CTGCCACCCAGAAGACTGTG-3'	5'-GGTCCTCAGTGTAGCCCAAG-3'

Off-target standard assays

Methods employed in this study have been adapted from the scientific literature to maximize reliability and reproducibility. Reference standards were run as an integral part of each assay to ensure the validity of the results obtained. Radioligand binding Assays were performed under conditions described in the accompanying "Methods" section of this report by **Eurofins Panlabs, Inc** (Taipei, Taiwan, ROC).

***hERG* patch-clamp assay**

BPRCX807 were tested in the *hERG* (*hERG*-CHO, automated patch-clamp) assay and determined at Eurofins Panlabs (Missouri, USA). The experiment was accepted in accordance with **Eurofins** validation Standard Operating Procedure.

Selectivity Screening of various chemokine receptors

The results were performed by **Eurofins DiscoverX** (Fremont, California, USA). The

PathHunter® β-Arrestin assay was performed in a homogenous, non-imaging assay format using a technology developed by DiscoverX called Enzyme Fragment Complementation (EFC) with β-galactosidase (β-Gal) as the functional reporter. The enzyme is split into two inactive complementary portions: a small peptide, called ProLink™ (PK) and a larger protein, called Enzyme Acceptor (EA). PK and EA are then expressed as fusion proteins in the cell, with PK fused to the GPCR of interest, and EA fused to β-arrestin. When the receptor is activated and β-arrestin is recruited to the receptor, PK and EA complementation occurs, restoring β-Gal activity which is measured using chemiluminescent PathHunter® Detection Reagents. For antagonist determination, cells were pre-incubated with BPRCX807 followed by indicated chemokine challenge. Intermediate dilution of sample stocks was performed to generate 5× sample in assay buffer. 5 μL of 5× sample was added to cells and incubated at 37 °C or room temperature for 30 minutes. Vehicle concentration was 1%. 5 μL of indicated chemokine in assay buffer was added to the cells and incubated at 37 °C or room temperature for 90 minutes to detect the effect of BPRCX807 on indicated receptors.

Statistics

All statistical analyses were performed with Graph Pad Prism software. According to the data distribution, the multiple *t*-test or the Mann-Whitney *U*-test was used. *p*-values smaller than 0.05 were considered to indicate statistical significance for all tests.

References

[1] C.-H. Wu, J.-S. Song, H.-H. Kuan, S.-H. Wu, M.-C. Chou, J.-J. Jan, L. K. Tsou, Y.-Y. Ke, C.-T. Chen, K.-C. Yeh, S.-Y. Wang, T.-K. Yeh, C.-T. Tseng, C.-L. Huang, M.-H. Wu, P.-C. Kuo, C.-J. Lee, K.-S. Shia, *J. Med. Chem.* **2018**, *61*, 818-833.

[2] S. A. Hermitage, K. S. Cardwell, T. Chapman, J. W. B. Cooke, R. Newton, *Org Process Res Dev* **2001**, *5*, 37-44.

[3] W. Kim, J. Seong, H. J. Oh, W. S. Koom, K.-J. Choi, C.-O. Yun, *J. Radiat. Res.* **2011**, *52*, 646-654.

[4] H. J. C. Berendsen, D. van der Spoel, R. van Drunen, *Comput. Phys. Commun.* **1995**, *91*, 43-56.

[5] A. W. Schuttelkopf, D. M. F. van Aalten, *Acta Crystallogr. D* **2004**, *60*, 1355-1363.

[6] S.-W. Chiu, S. A. Pandit, H. L. Scott, E. Jakobsson, *J. Phys. Chem. B* **2009**, *113*, 2748-2763.

[7] B. Hess, H. Bekker, H. J. C. Berendsen, J. G. E. M. Fraaije, *J. Comput. Chem.* **1997**, *18*, 1463-1472.

[8] Y. Huang, J. Yuan, E. Righi, W. S. Kamoun, M. Ancukiewicz, J. Nezivar, M. Santosuosso, J. D. Martin, M. R. Martin, F. Vianello, P. Leblanc, L. L. Munn, P. Huang, D. G. Duda, D. Fukumura, R.K. Jain and M. C. Poznansky, *Proc. Natl. Acad. Sci. U.S.A.* **2012**, *109*, 17561-17566.

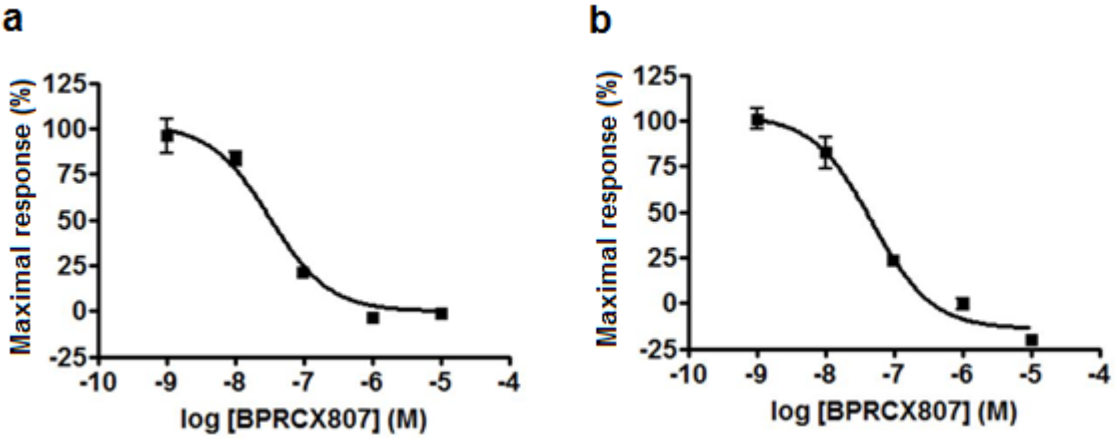

Figure S1. Binding affinity and chemotaxis inhibition assay of BPRCX807. (a) Binding affinity (IC_{50}) of BPRCX807 was determined by 50% inhibition of radioligand [^{125}I]CXCL12 binding to hCXCR4-transfected HEK293T membrane; (b) CXCL12-induced chemotaxis inhibition assay was performed to measure the inhibitory activity of BPRCX807 using CCRF-CEM cell line. Values represent the mean ± SD of at least three independent experiments.

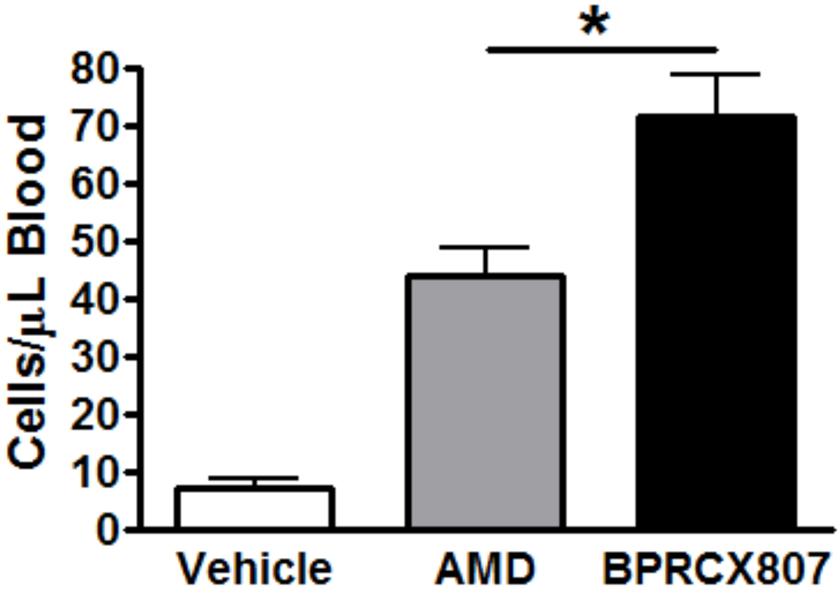

Figure S2. $CXCR4^{+}CD34^{+}$ stem cell mobilization. C57BL/6 male mice were subcutaneously administered with vehicle, AMD3100 (6 mg/kg), or BPRCX807 (6 mg/kg) 2 h after dosing, the peripheral blood was harvested for analyzing the number of $CXCR4^{+}CD34^{+}$ stem cells. The data are the mean values ± SEM (n = 3/group). Statistical analysis was performed by *t*-test: *$p < 0.05$ between AMD3100 and BPRCX807.

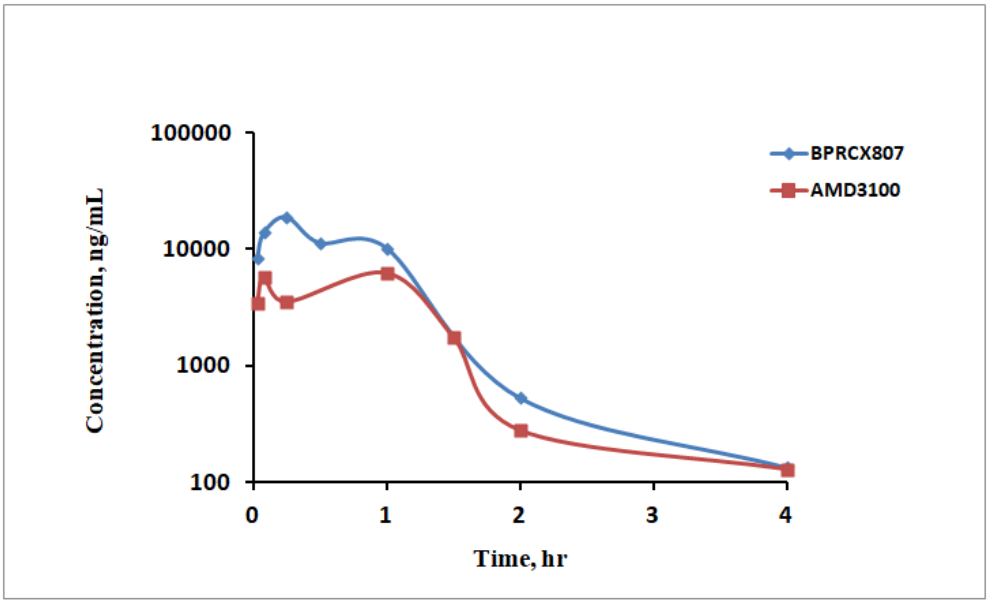

Figure S3. The plasma concentration profile (AUC) of BPRCX807 and AMD3100. Y-axis is on a log scale; pharmacokinetic analysis following SC (6 mg/kg) in C57BL/6 mice for each time point n = 3.

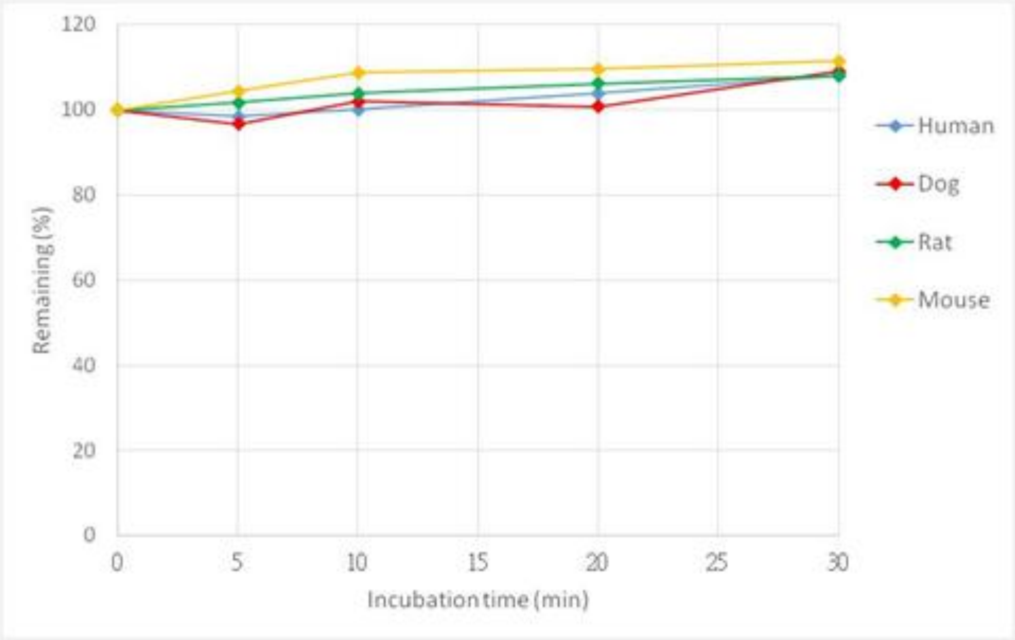

Figure S4. The metabolic stabilities of BPRCX807 in liver microsomes. BPRCX807 is metabolically stable in mouse, rat, dog and human.

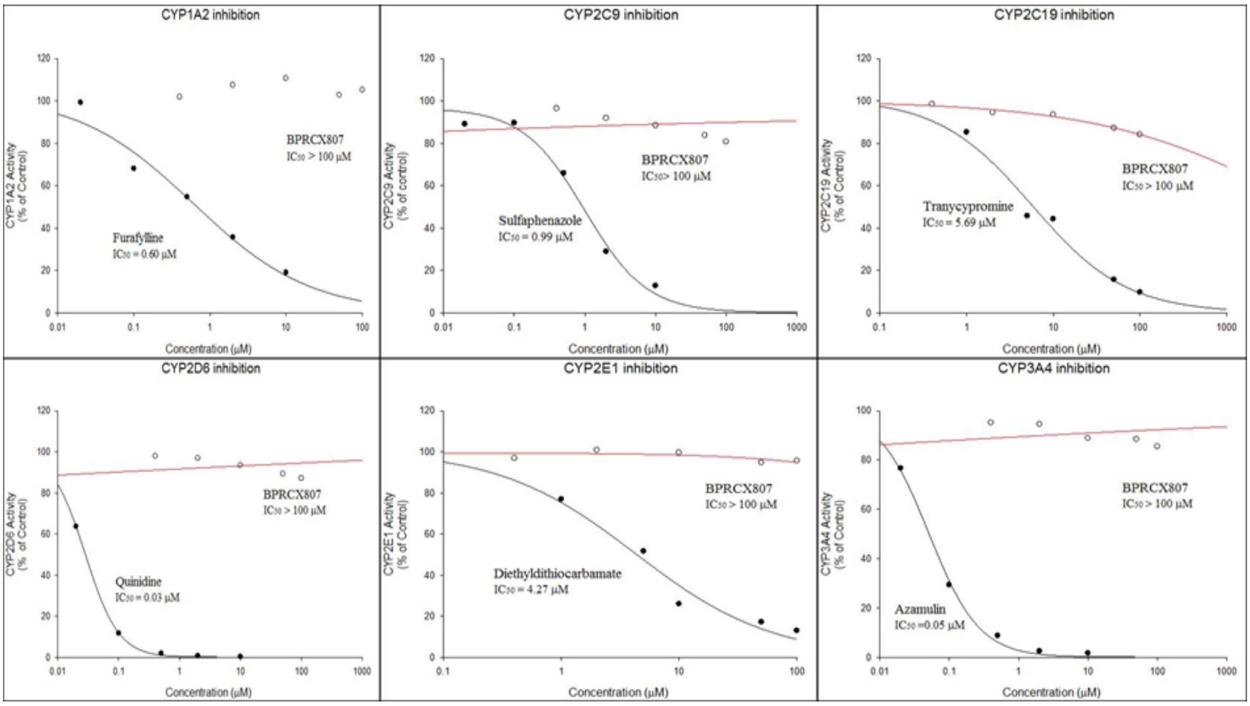

Figure S5. Inhibitory values (IC_{50}) of BPRCX807 on 6 human liver P450 isozymes.

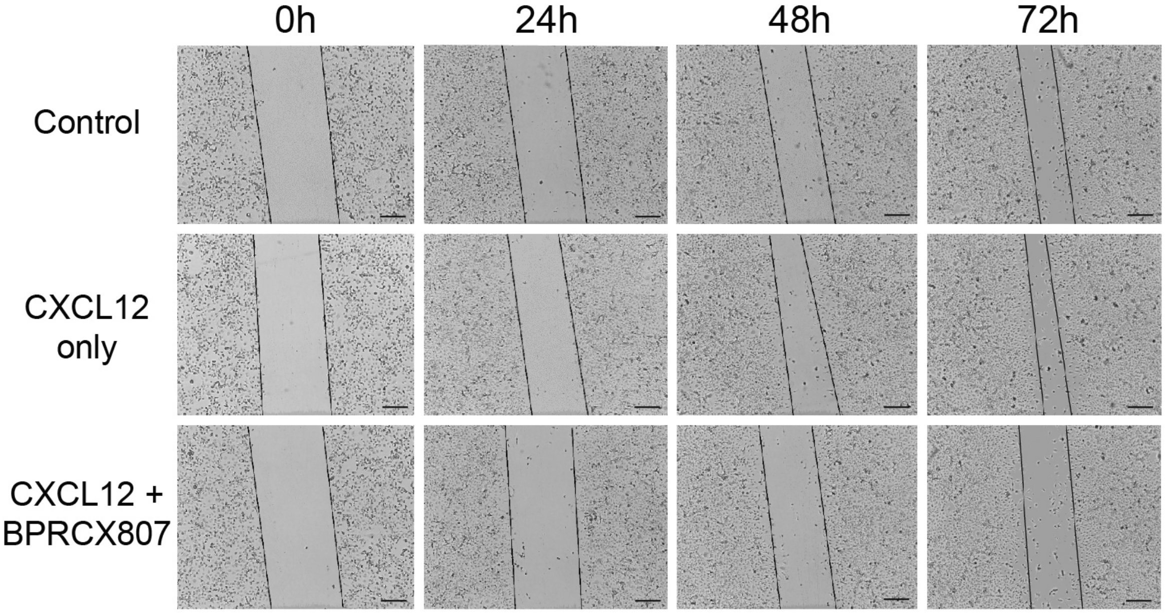

Figure S6. BPRCX807 suppresses CXCL12/CXCR4 axis-mediated migration of HCC cells. Representative images from wound healing migration assays using HCA-1 cells incubated for 24, 48, and 72 h after treatment with CXCL12 (100 ng/mL) and BPRCX807 (10 μM). Scale bar = 200 μm.

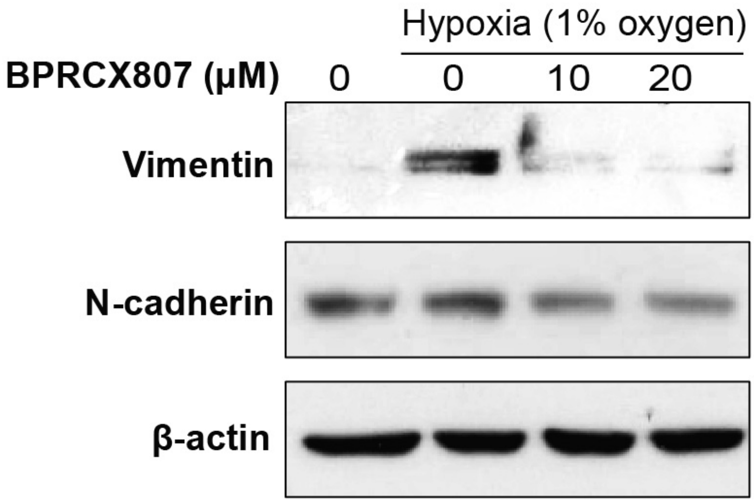

Figure S7. BPRCX807 inhibits the EMT phenotype of HCA-1 cells under hypoxic conditions (1% oxygen). The protein levels of mesenchymal markers (vimentin and N-cadherin) were determined by western blotting 48 h after treatment with **BPRCX807** at different doses.

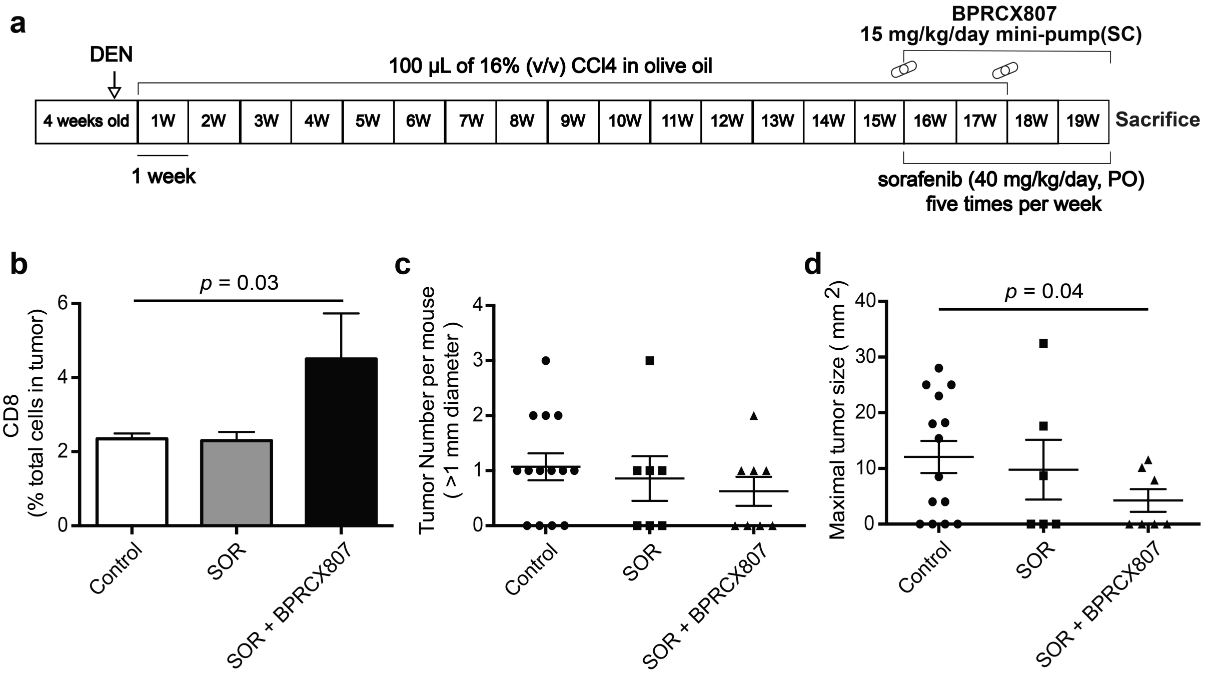

Figure S8. DEN/CCl$_4$-induced liver fibrosis associated HCC model. (a) Experimental protocol. (b) Analysis of CD8$^+$ T cells (n = 4-6). (c) Liver tumor nodule counts (>1 mm) per mouse. (d) Measurement of tumor size. The data are the mean value ± S.E.M..

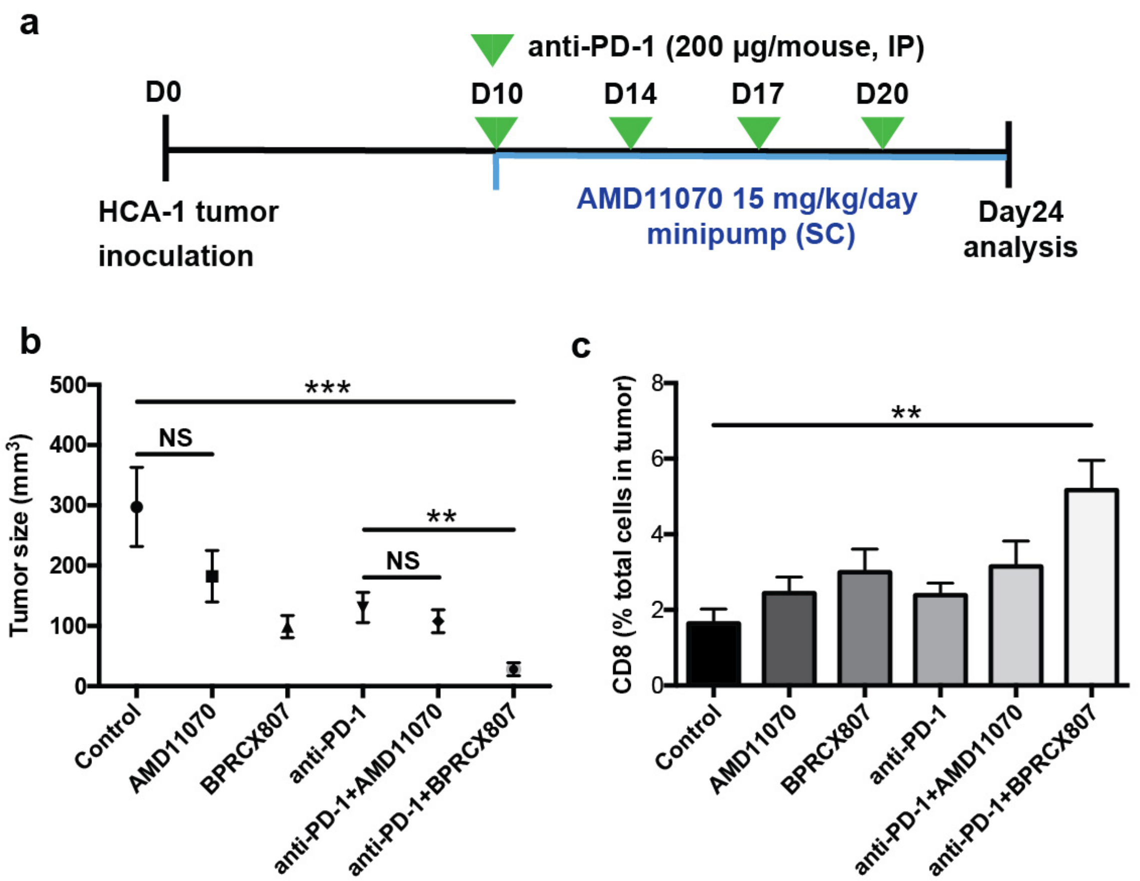

Figure S9. Synergistic effects of AMD11070 with immune therapy in the orthotopic HCA-1 model. (a) Treatment protocol. Ten days after implantation of HCC cells, mice were treated with **AMD11070** (15 mg/kg/day, SC) by minipump from day 10 to day 24. Anti-PD-1 Ab (200 μg/mouse) was injected IP on days 10, 14, 17, and 20 after tumor implantation. (b) Tumor sizes (n = 8-10). (c) T cells (n = 4-8) was shown in $CD8^+$ T cells. The data are the mean value ± S.E.M.. $^*p < 0.05$, $^{**}p < 0.01$, and $^{***}p < 0.001$.

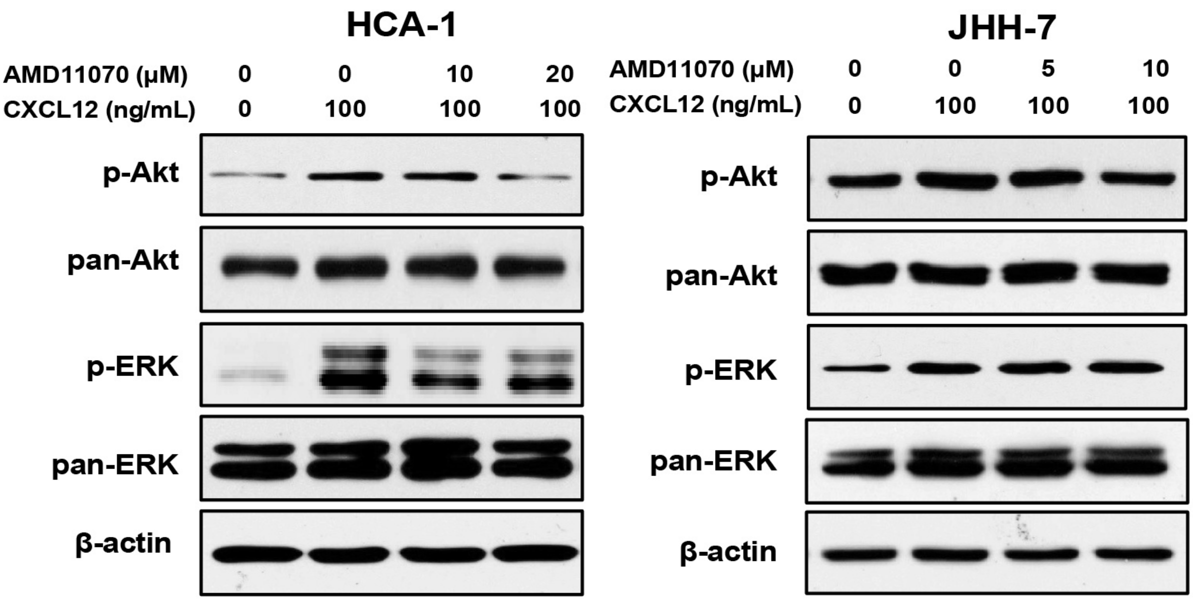

Figure S10. AMD11070 moderately inhibited CXCL12-mediated cell signaling pathways. Western blot analysis of phospho-ERK and phospho-AKT in HCA-1 and JHH-7 cells.

^{1}H NMR of Linker 1

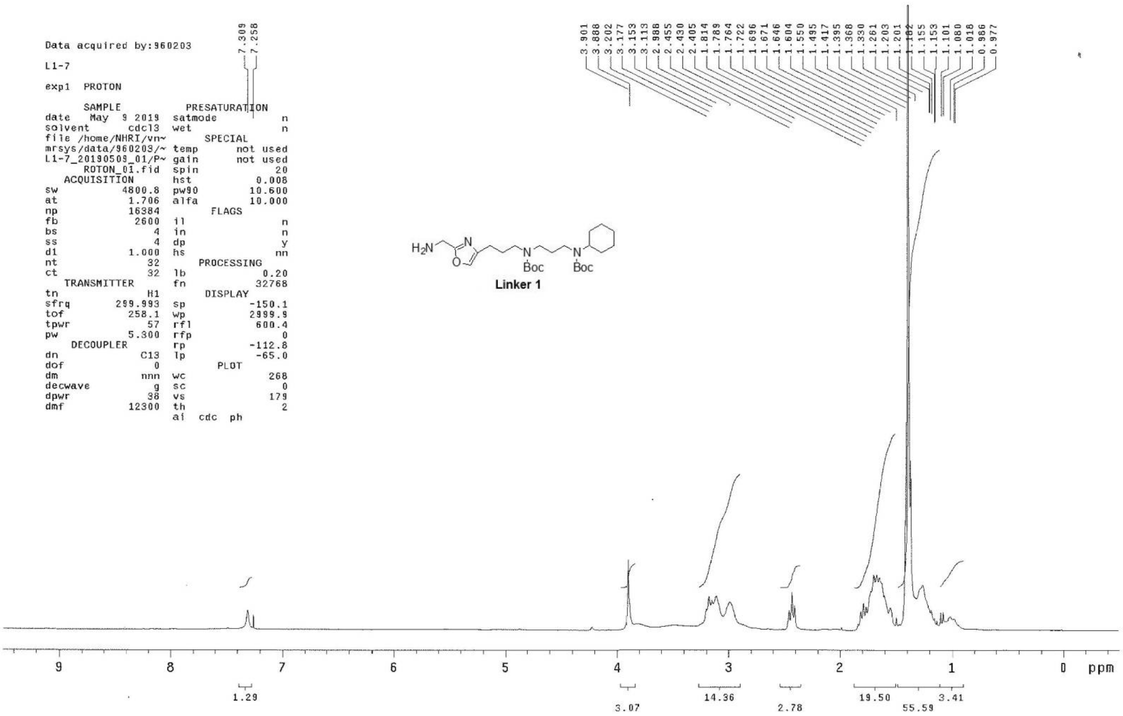

^{1}H NMR spectrum of Linker 1

^{13}C NMR of Linker 1

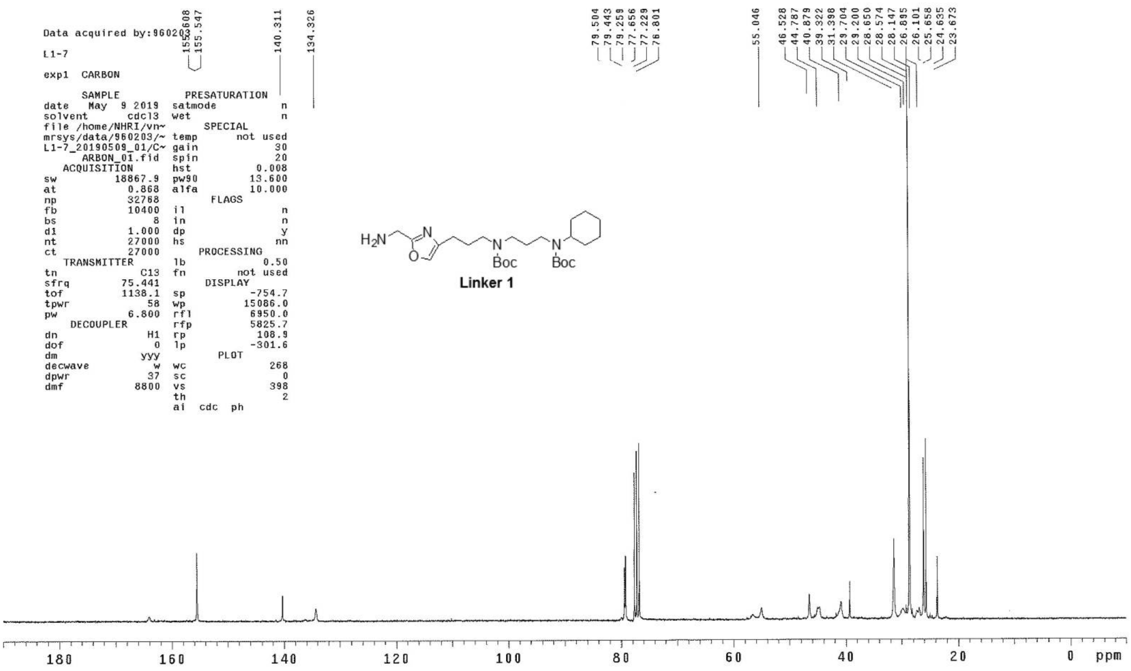

^{13}C NMR spectrum of Linker 1

^{1}H NMR of compound 1

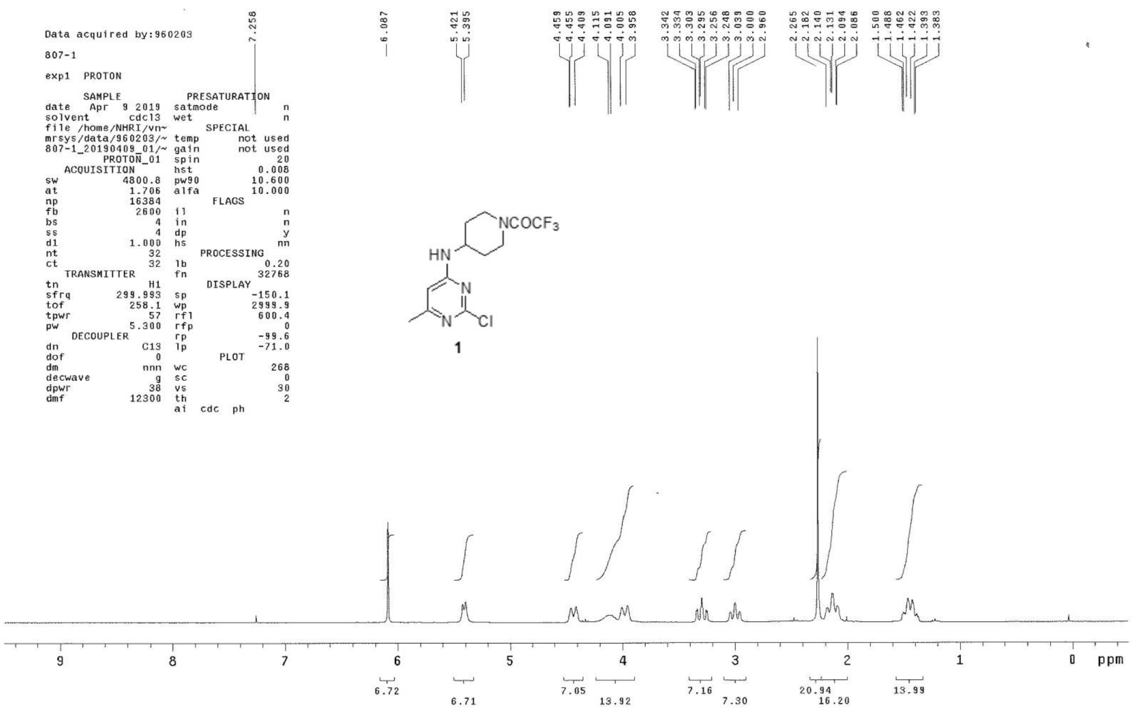

^{1}H NMR spectrum of compound 1

^{13}C NMR of compound 1

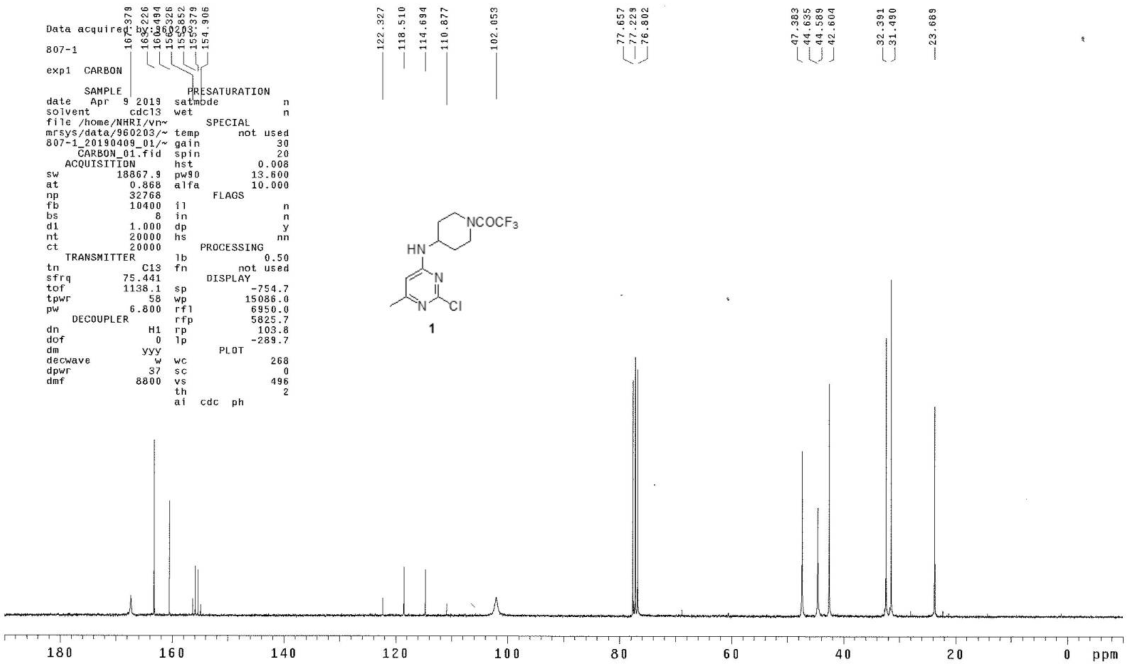

^{13}C NMR spectrum of compound 1

^{1}H NMR of compound 2

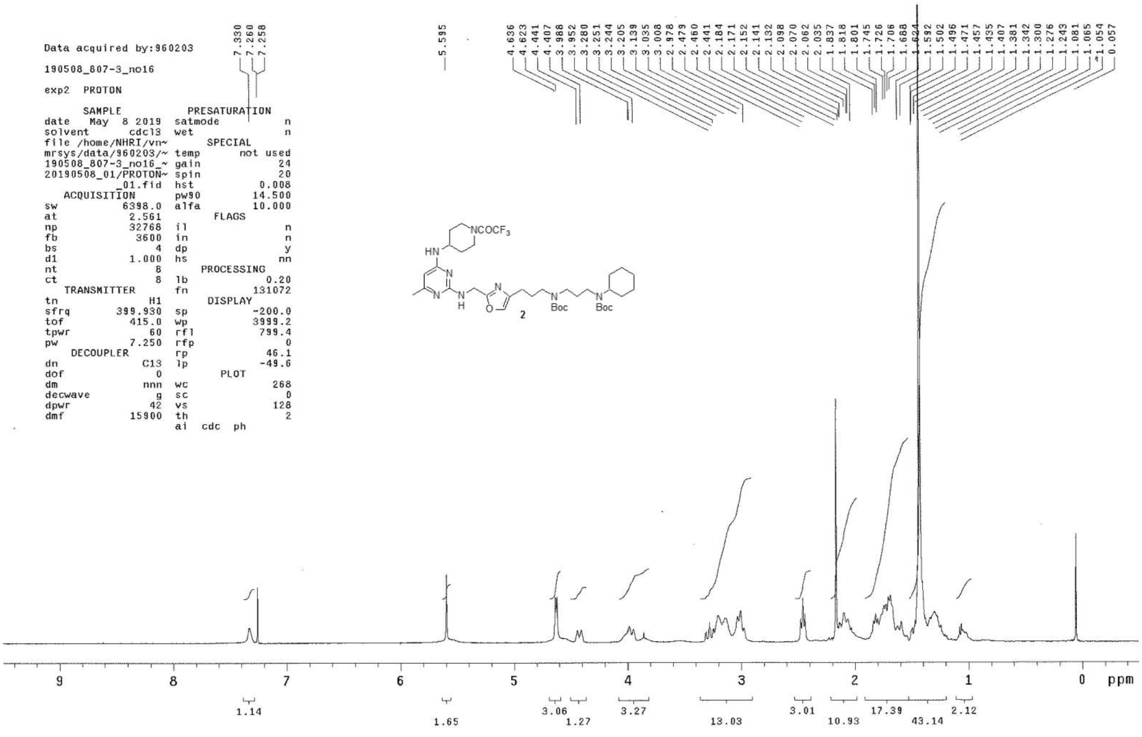

^{1}H NMR spectrum of compound 2

^{13}C NMR of compound 2

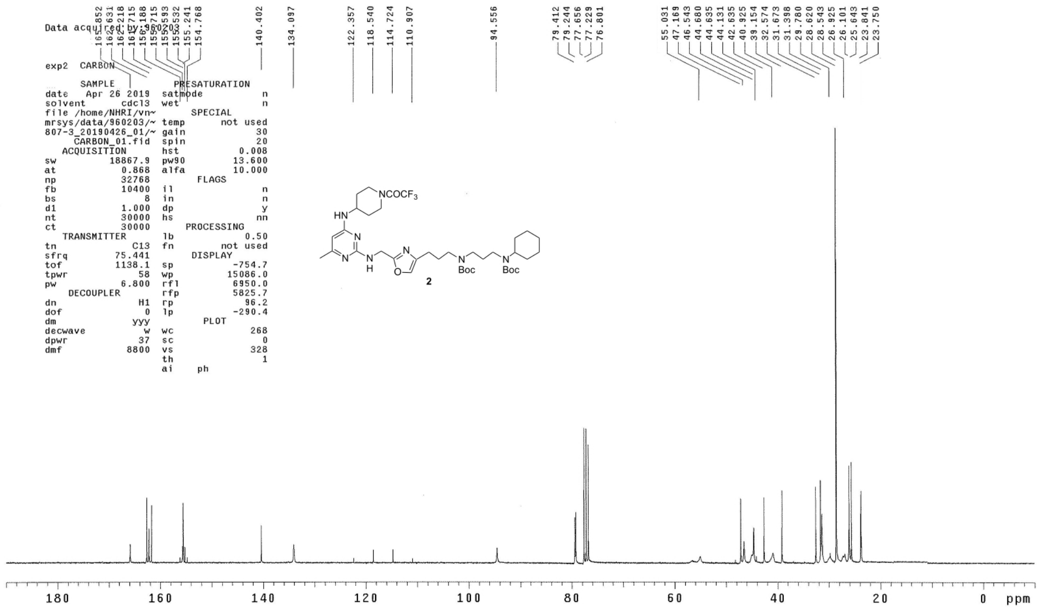

^{13}C NMR spectrum of compound 2

^{1}H NMR of compound 3

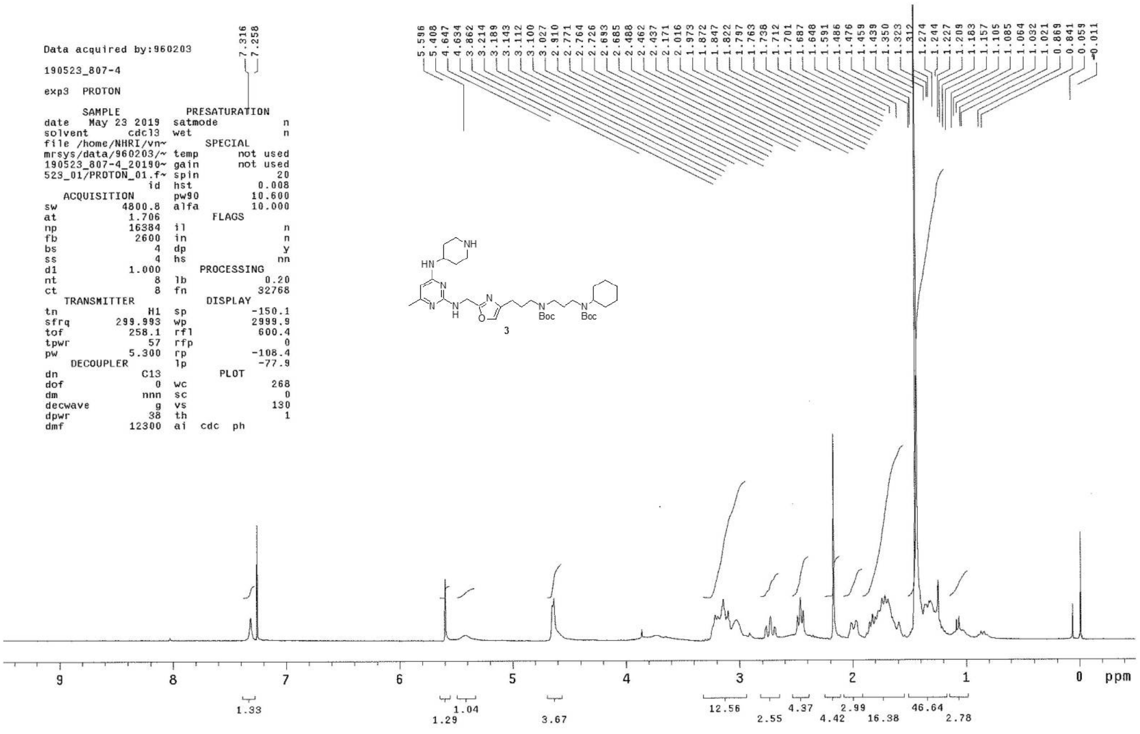

^{1}H NMR spectrum of compound 3

^{13}C NMR of compound 3

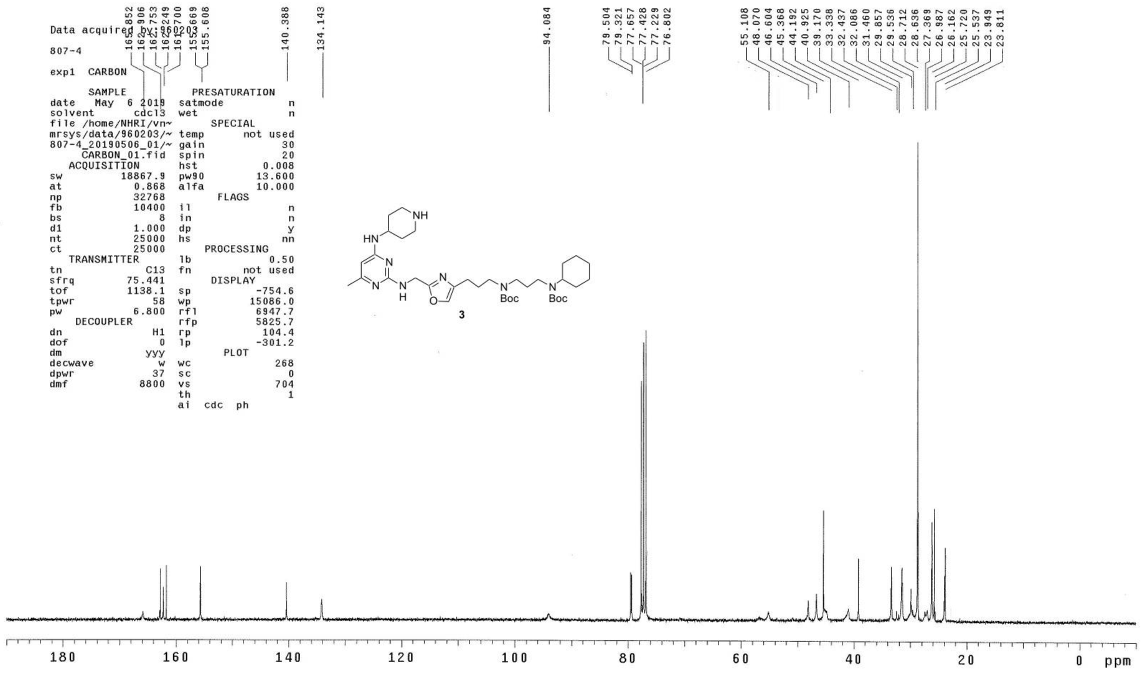

^{13}C NMR spectrum of compound 3

^{1}H NMR of compound 4

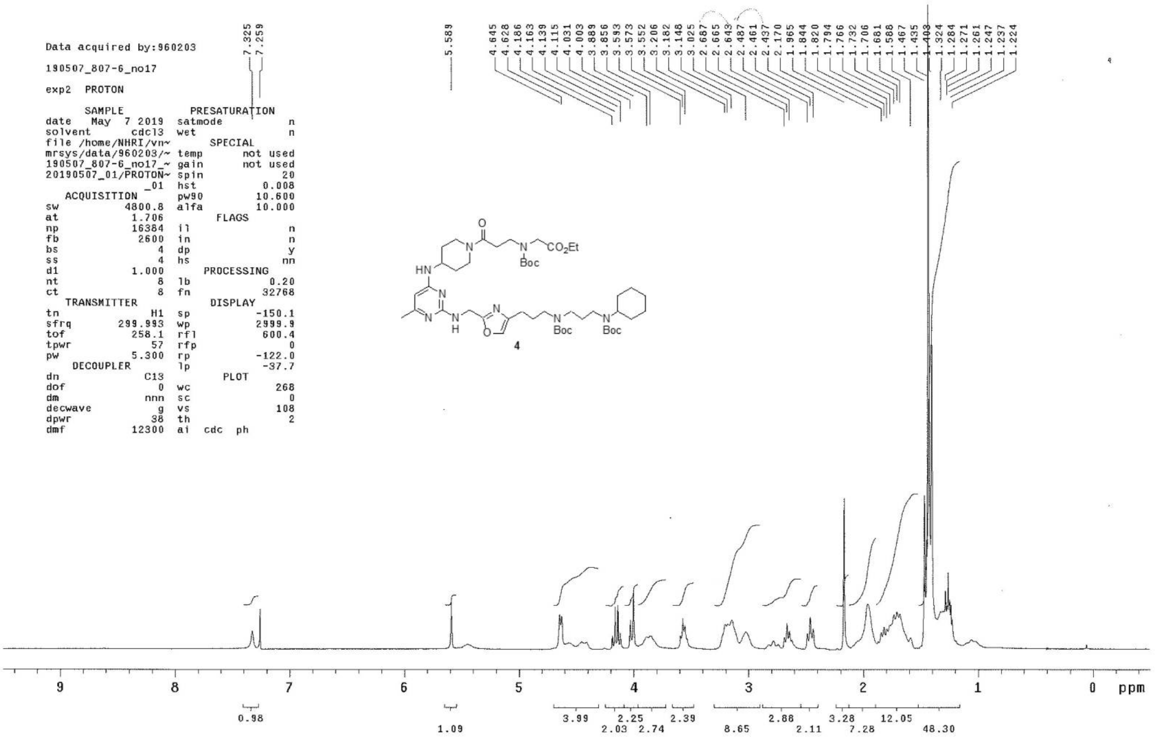

^{1}H NMR spectrum of compound 4

^{13}C NMR of compound 4

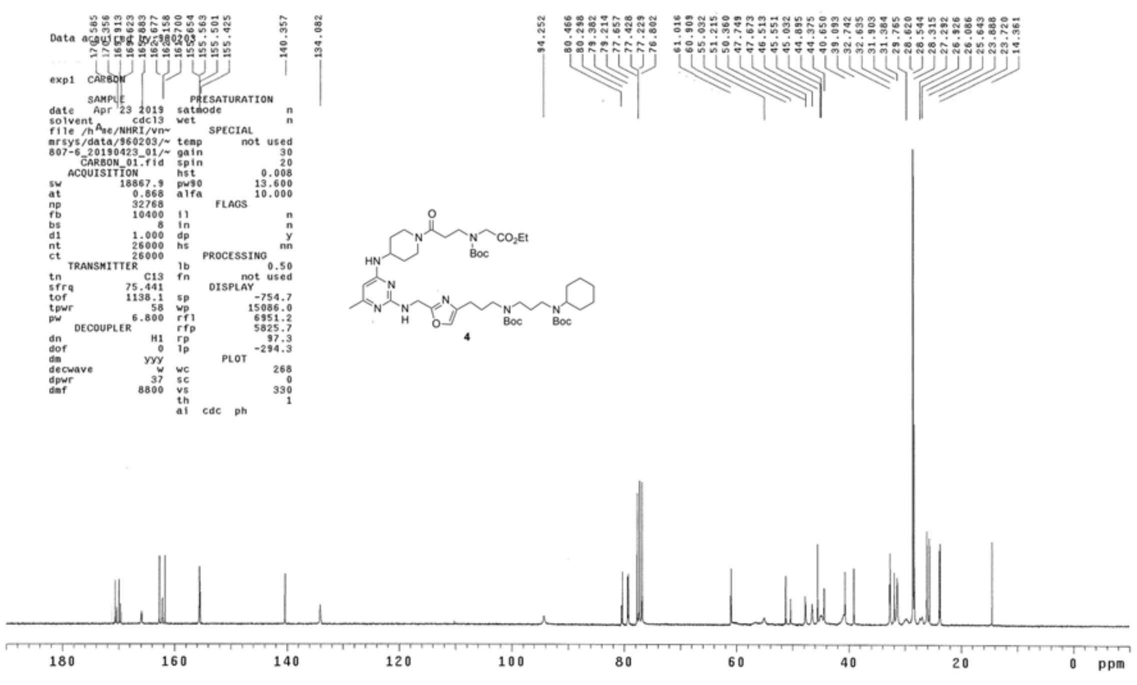

^{13}C NMR spectrum of compound 4

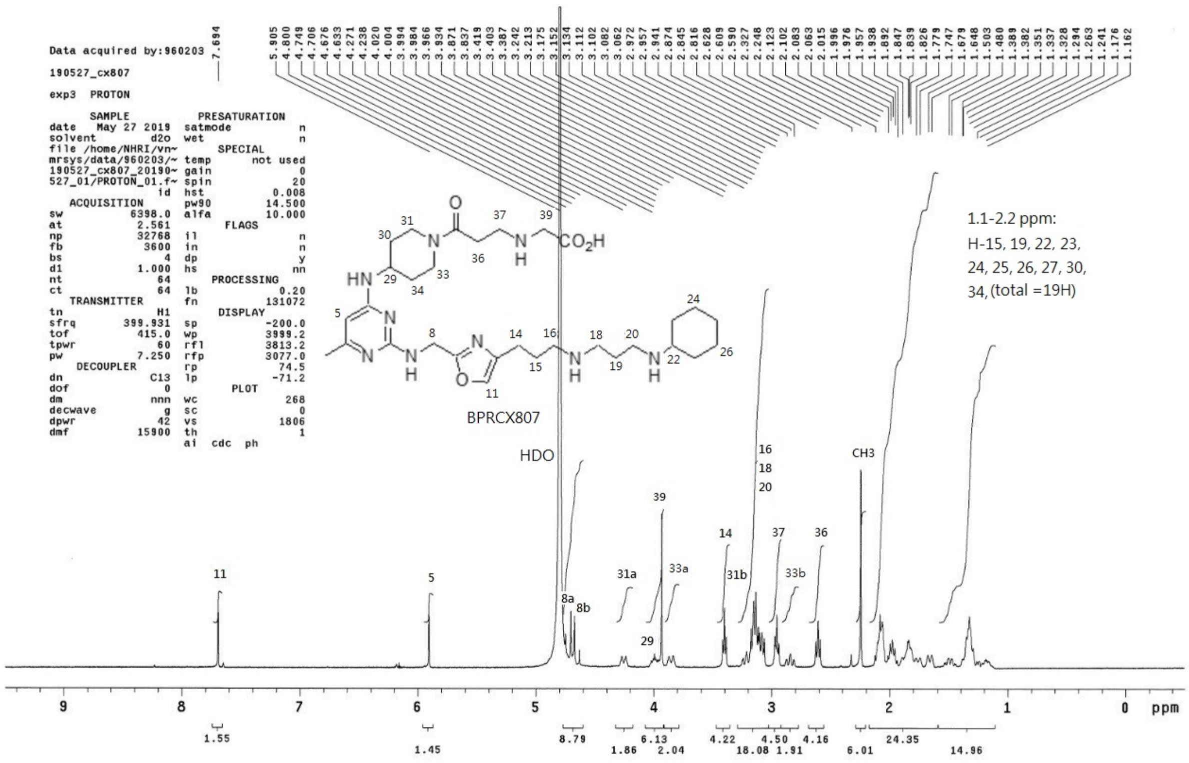

^{1}H NMR spectrum of BPRCX807

^{13}C NMR of BPRCX807

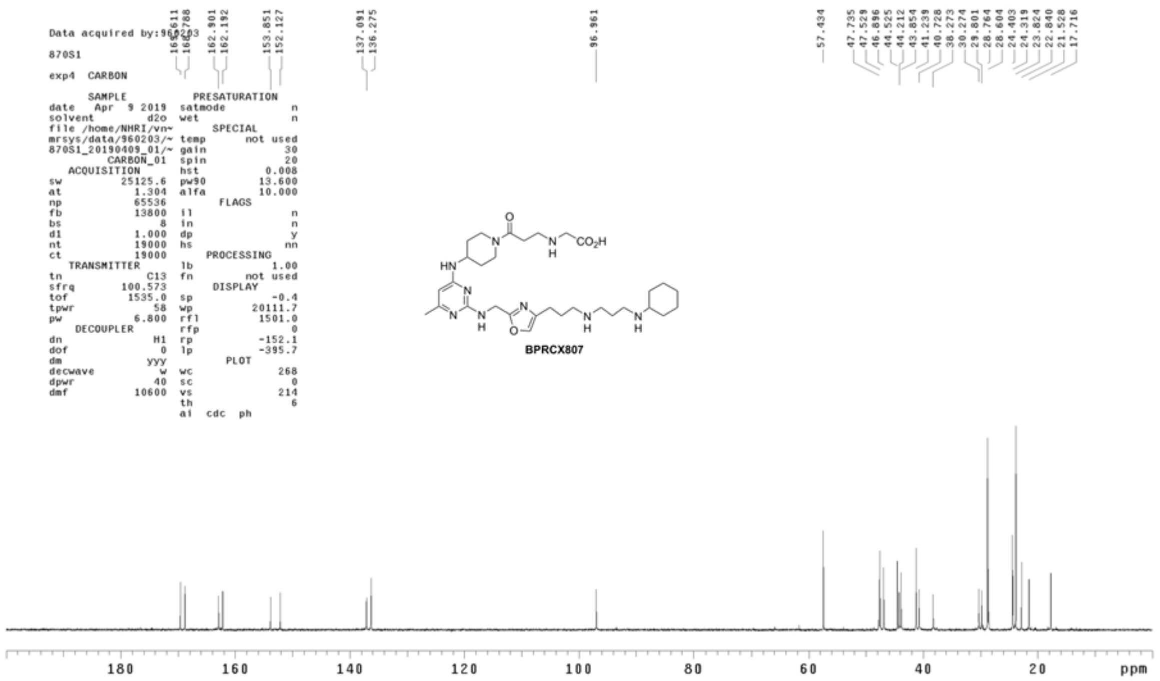

^{13}C NMR spectrum of BPRCX807

Diagram I. Purity of BPRCX807 determined by analytic Method 1.

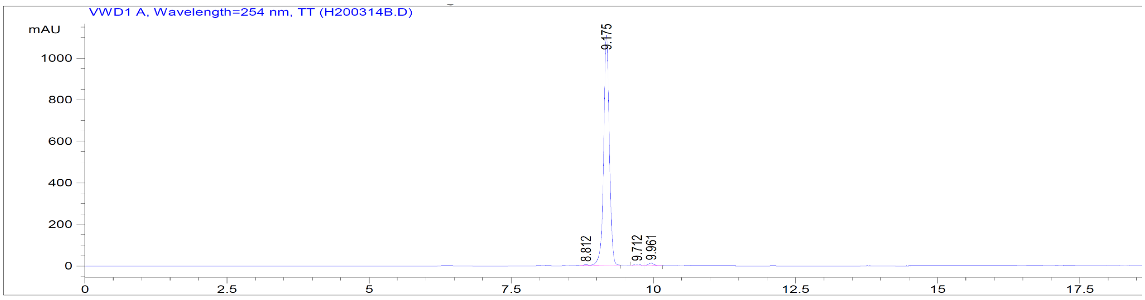


```
=====================================================================
                         Area Percent Report
=====================================================================

Sorted By             :      Signal
Multiplier            :      1.0000
Dilution              :      1.0000
Use Multiplier & Dilution Factor with ISTDs

Signal 1: VWD1 A, Wavelength=254 nm, TT
```

Peak #	RetTime [min]	Type	Width [min]	Area mAU *s	Height [mAU]	Area %
1	8.812	PV	0.0970	30.53537	5.24633	0.3915
2	9.175	VB	0.1153	7650.59668	1106.34131	98.0922
3	9.712	PV	0.0973	43.54694	6.99813	0.5583
4	9.961	VB	0.0995	74.71322	11.43210	0.9579

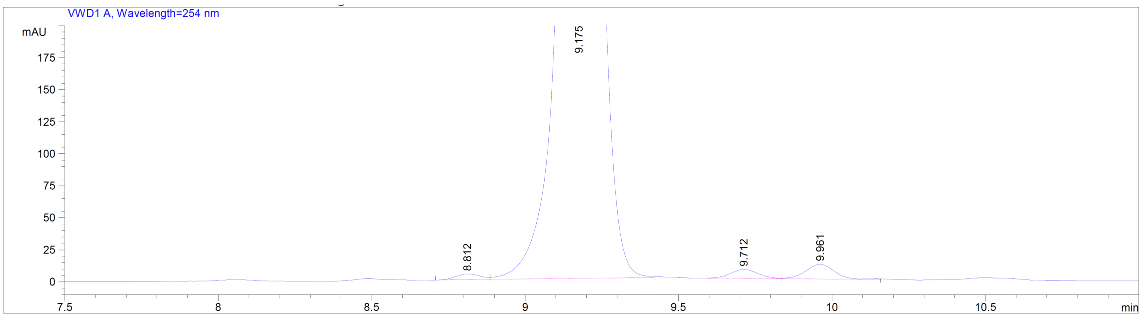

Method 1: A gradient system mixed with two eluting solvents A (water with 0.1% TFA) and B (MeOH) using a flow rate of 1 mL/min starting with 2 min at 10% solvent B, followed by a 4 min gradient of 10-50% solvent B, followed by a 10 minute gradient of 50-90% solvent B, followed by 9 min at 90% solvent B.

Diagram II. Purity of BPRCX807 determined by analytic Method 2.

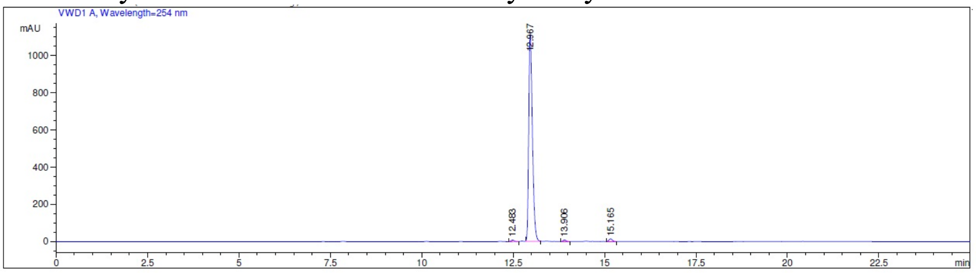

Area Percent Report

Signal 1: VWD1 A, Wavelength=254 nm

Peak #	RetTime [min]	Type	Width [min]	Area mAU *s	Height [mAU]	Area %
1	12.483	PP	0.0905	48.14585	8.17517	0.6021
2	12.967	BB	0.1062	7812.17969	1118.92590	97.6895
3	13.906	PP	0.0877	46.57333	8.25052	0.5824
4	15.165	BB	0.0972	90.05299	14.22255	1.1261

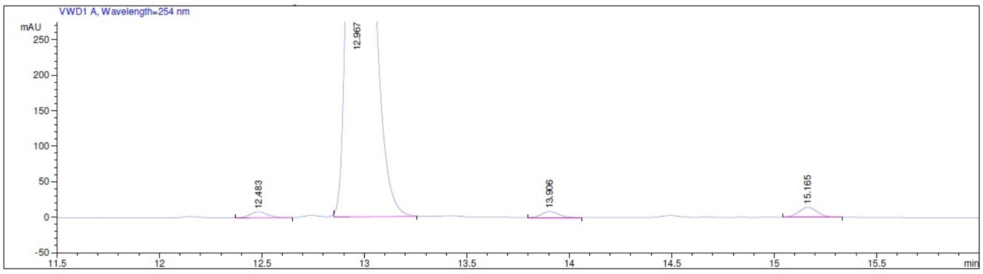

Method 2: A gradient system mixed with two eluting solvents A (water with 0.1% TFA) and B (MeOH) using a flow rate of 1 mL/min starting with 2 minute at 10% solvent B, followed by a 4 minute gradient of 10-20% solvent B, followed by a 10 minute gradient of 20-60% solvent B, followed by 9 minutes at 60% solvent B.

Table S1. Organ weight measurement of BPRCX807

	**Vehicle, SC, n=5**	**BPRCX807, 50 mg/kg, SC, n=5**	**P value**
(g)	Mean ± S.D.		
Body Weight	326.8 ± 14.1	311.8 ± 15.7	0.1490
Liver	16.4419 ± 1.5350	14.3954 ± 2.0460	0.1114
Liver/B.W.	0.0503 ± 0.0045	0.0460 ± 0.0044	0.1642
Spleen	0.6643 ± 0.0680	0.6487 ± 0.1648	0.8492
Spleen/B.W.	0.0020 ± 0.0002	0.0021 ± 0.0004	0.8785
Kidney (2)	3.1582 ± 0.4988	2.9826± 0.4737	0.5838
Kidneys/B.W.	0.0097 ± 0.0014	0.0095 ± 0.0011	0.8781
Adrenal Glands (2)	0.0612 ± 0.0077	0.0612 ± 0.0083	0.9908
Adrenal Glands/B.W.	0.00019 ± 0.00003	0.00020 ± 0.00003	0.6493
Thymus	0.7416 ± 0.0982	0.6705 ± 0.2266	0.5375
Thymus/B.W.	0.0023 ± 0.0002	0.0021 ± 0.0006	0.6446
Heart	1.2836 ± 0.1619	1.1704 ± 0.1340	0.2629
Heart/B.W.	0.0039 ± 0.0004	0.0038 ± 0.0004	0.5197
Lung	1.6794 ± 0.3847	1.6847 ± 0.1313	0.9776
Lung/B.W.	0.0051 ± 0.0012	0.0054 ± 0.0005	0.6410
Testes (2)	2.9324 ± 0.1515	2.9421 ± 0.2476	0.9420
Testes/B.W.	0.0090 ± 0.0008	0.0094 ± 0.0006	0.3401

Table S2. Hematology analysis of BPRCX807

	**Vehicle, SC, n=5**	**BPRCX807, 50 mg/kg, SC, n=5**	**P value**
	Mean ± S.D.		
White blood cells (10^3/μL)	8.15 ± 4.03	10.75 ± 6.09	0.4486
Neutrophil (10^3/μL)	2.67 ± 1.83	3.75 ± 2.13	0.4182
Lymphocyte (10^3/μL)	4.96 ± 2.01	6.36 ± 3.70	0.4794
Monocyte (10^3/μL)	0.49 ± 0.23	0.63 ± 0.36	0.4939
Eosinophil (10^3/μL)	0.01 ± 0.01	0.01 ± 0.01	0.7924
Basophil (10^3/μL)	0.002 ± 0.004	0.002 ± 0.004	1.0000
Neutrophil %	29.80 ± 8.10	35.11 ± 5.51	0.2602
Lymphocyte %	63.80 ± 8.35	58.95 ± 6.42	0.3333
Monocyte %	6.22± 1.46	5.82 ± 1.11	0.6423
Eosinophil %	0.14 ± 0.13	0.11 ± 0.05	0.6773
Basophil %	0.04 ± 0.05	0.01 ± 0.02	0.2495
Erythrocyte (10^6/μL)	6.47 ± 0.35	6.33 ± 0.45	0.5861
Hemoglobin (g/dL)	13.9 ± 1.0	13.8 ± 0.9	0.8190
Hematocrit (g/dL)	41.2 ± 1.3	41.4 ± 3.5	0.9077
Mean corpuscle volume (fL)	63.7 ± 1.9	65.4 ± 2.7	0.2876
Mean corpuscle hemoglobin (pg)	21.5 ± 0.7	21.8 ± 1.0	0.6059
Mean corpuscle hemoglobin concentration (MCHC) (g/dL)	33.7 ± 1.4	33.3 ± 1.5	0.6681
Red blood cell distribution width (%)	15.1 ± 0.6	15.1 ± 0.5	0.9570
Platelet (10^3/μL)	1195 ± 207	1327± 239	0.3758
Mean platelet volume (fL)	6.4 ± 0.3	6.4 ± 0.1	1.0000

Table S3. Biochemistry analysis of BPRCX807

	**Vehicle, SC, n=5**	**BPRCX807, 50 mg/kg, SC, n=5**	**P value**
	Mean ± S.D.		
Glutamate oxaloacetate transaminase (GOT) (U/L)	78 ± 26	67 ± 7	0.4086
Glutamate pyruvate transaminase (GPT) (U/L)	37 ± 3	35 ± 5	0.4298
Lactate dehydrogenase (LDH) (U/L)	863 ± 1279	272 ± 132	0.3337
Alkaline Phosphatase (ALP) (U/L)	1133 ± 337	901 ± 60	0.1694
Blood Urea Nitrogen (BUN) (mg/dL)	16.2 ± 1.8	16.7 ± 2.1	0.6966
Creatinine (CRE) (mg/dL)	0.20 ± 0.07	0.22 ± 0.04	0.6075
Albumin (ALB) (g/dL)	3.9 ± 0.3	4.0 ± 0.2	0.4676

Table S4. 67 off-target standard assays of BPRCX807

Cat #	Assay Name	Batch*	Spec.	Rep.	Conc.	% Inh.
**Compound: CX807, PT #: 1218479**						
200510	Adenosine A_1	421425	hum	2	10 μM	2
200610	Adenosine A_{2A}	421426	hum	2	10 μM	4
200720	Adenosine A_3	421407	hum	2	10 μM	20
203100	Adrenergic α_{1A}	421531	rat	2	10 μM	13
203200	Adrenergic α_{1B}	421532	rat	2	10 μM	10
203400	Adrenergic α_{1D}	421409	hum	2	10 μM	20
203630	Adrenergic α_{2A}	421427	hum	2	10 μM	16
204010	Adrenergic β_1	421491	hum	2	10 μM	4
204110	Adrenergic β_2	421428	hum	2	10 μM	1
206000	Androgen (Testosterone)	421561	hum	2	10 μM	1
212520	Bradykinin B_1	421593	hum	2	10 μM	62
212620	Bradykinin B_2	421424	hum	2	10 μM	-7
214510	Calcium Channel L-Type, Benzothiazepine	421527	rat	2	10 μM	12
214600	Calcium Channel L-Type, Dihydropyridine	421415	rat	2	10 μM	10
216000	Calcium Channel N-Type	421585	rat	2	10 μM	35
217030	Cannabinoid CB_1	421534	hum	2	10 μM	5
219500	Dopamine D_1	421729	hum	2	10 μM	7
219700	Dopamine D_{2S}	421451	hum	2	10 μM	13
219800	Dopamine D_3	421564	hum	2	10 μM	58
219900	Dopamine $D_{4.2}$	421461	hum	2	10 μM	6
224010	Endothelin ET_A	421459	hum	2	10 μM	0
224110	Endothelin ET_B	421414	hum	2	10 μM	4
225510	Epidermal Growth Factor (EGF)	421529	hum	2	10 μM	-6
226010	Estrogen ERα	421462	hum	2	10 μM	-8
226600	$GABA_A$, Flunitrazepam, Central	421435	rat	2	10 μM	3
226500	$GABA_A$, Muscimol, Central	421464	rat	2	10 μM	-9
228610	$GABA_{B1A}$	421723	hum	2	10 μM	-1
232030	Glucocorticoid	421493	hum	2	10 μM	0
232710	Glutamate, Kainate	421468	rat	2	10 μM	-9
232810	Glutamate, NMDA, Agonism	421465	rat	2	10 μM	0
232910	Glutamate, NMDA, Glycine	421466	rat	2	10 μM	-5
233000	Glutamate, NMDA, Phencyclidine	421430	rat	2	10 μM	-15
239610	Histamine H_1	421537	hum	2	10 μM	0
239710	Histamine H_2	421488	hum	2	10 μM	-7

Note: Items meeting criteria for significance (≥50% stimulation or inhibition) are highlighted.
* Batch: Represents compounds tested concurrently in the same assay(s).
ham=Hamster; hum=Human

Cat #	Assay Name	Batch*	Spec.	Rep.	Conc.	% Inh.
239820	Histamine H_3	421431	hum	2	10 μM	18
241000	Imidazoline I_2, Central	421538	rat	2	10 μM	16
250460	Leukotriene, Cysteinyl $CysLT_1$	421485	hum	2	10 μM	1
251600	Melatonin MT_1	421486	hum	2	10 μM	-7
252610	Muscarinic M_1	421401	hum	2	10 μM	11
252710	Muscarinic M_2	421402	hum	2	10 μM	31
252810	Muscarinic M_3	421403	hum	2	10 μM	6
257010	Neuropeptide Y Y_1	421419	hum	2	10 μM	11
257110	Neuropeptide Y Y_2	421420	hum	2	10 μM	5
258590	Nicotinic Acetylcholine	421539	hum	2	10 μM	3
258700	Nicotinic Acetylcholine α1, Bungarotoxin	421489	hum	2	10 μM	12
260130	Opiate δ_1 (OP1, DOP)	421406	hum	2	10 μM	7
260210	Opiate κ (OP2, KOP)	421477	hum	2	10 μM	27
260410	Opiate μ (OP3, MOP)	421543	hum	2	10 μM	8
264500	Phorbol Ester	421435	mouse	2	10 μM	3
265010	Platelet Activating Factor (PAF)	421652	hum	2	10 μM	23
265600	Potassium Channel [K_{ATP}]	421547	ham	2	10 μM	2
265900	Potassium Channel hERG	421548	hum	2	10 μM	12
268420	Prostanoid EP_4	421549	hum	2	10 μM	5
268700	Purinergic P2X	421524	rabbit	2	10 μM	3
268810	Purinergic P2Y	421596	rat	2	10 μM	2
270000	Rolipram	421413	rat	2	10 μM	2
271110	Serotonin (5-Hydroxytryptamine) 5-HT_{1A}	421473	hum	2	10 μM	0
271700	Serotonin (5-Hydroxytryptamine) 5-HT_{2B}	421550	hum	2	10 μM	8
271910	Serotonin (5-Hydroxytryptamine) 5-HT_3	421437	hum	2	10 μM	3
278110	Sigma σ_1	421551	hum	2	10 μM	5
279510	Sodium Channel, Site 2	421552	rat	2	10 μM	-3
255520	Tachykinin NK_1	421472	hum	2	10 μM	3
285900	Thyroid Hormone	421482	rat	2	10 μM	-9
220320	Transporter, Dopamine (DAT)	421492	hum	2	10 μM	48
226400	Transporter, GABA	421594	rat	2	10 μM	1
204410	Transporter, Norepinephrine (NET)	421553	hum	2	10 μM	25
274030	Transporter, Serotonin (5-Hydroxytryptamine) (SERT)	421445	hum	2	10 μM	10

Note: Items meeting criteria for significance (≥50% stimulation or inhibition) are highlighted.
* Batch: Represents compounds tested concurrently in the same assay(s).
ham=Hamster; hum=Human

Table S5. *hERG* patch-clamp assay of BPRCX807

Tested [C]	100 μM	10 μM	1 μM
Inhibition%	17.4%	9.0%	13.8%

Table S6. Selectivity screening of various chemokine receptors

assay target	efficacy (%)[a]	assay target	efficacy (%)[a]
CCR1	0	CCR10	0
CCR2	-3	CX3CR1	3
CCR3	4	CXCR1	10
CCR4	1	CXCR2	5
CCR5	1	CXCR3	6
CCR6	-8	CXCR4	101
CCR7	-6	CXCR5	-9
CCR8	-9	CXCR6	6
CCR9	-8	CXCR7	-20

[a]Percent inhibition was determined in the GPCR β-arrestin assay at a concentration of 10 μM and very weak inhibition (-20~10%) was observed for all tested chemokine receptors except CXCR4 (101%).
